# Adherence to American Cancer Society Guideline and Mortality in Men With Nonmetastatic Prostate Cancer

**DOI:** 10.1001/jamanetworkopen.2025.33922

**Published:** 2025-09-26

**Authors:** Valeria Elahy, Christina C. Newton, Marjorie L. McCullough, Lauren R. Teras, Clara Bodelon, Erika Rees-Punia, Caroline Y. Um, Laura Makaroff, Alpa V. Patel, Ying Wang

**Affiliations:** 1 Department of Population Science, American Cancer Society, Atlanta, Georgia; 2 Department of Cancer Prevention and Survivorship, American Cancer Society, Atlanta, Georgia

## Abstract

**Importance:**

Among men with nonmetastatic prostate cancer, prostate cancer–specific mortality (PCSM) is the leading cancer-related cause of death, and cardiovascular disease mortality (CVDM) is the most common non–cancer-related cause. The American Cancer Society (ACS) guideline for cancer survivors recommends avoiding obesity, engaging in regular physical activity, and following a healthy diet. While physical activity and body weight have been associated individually with lower mortality in patients with prostate cancer, the combined effect of all ACS guideline components remains unclear.

**Objective:**

To examine the association between postdiagnosis ACS guideline concordance and mortality in nonmetastatic prostate cancer survivors.

**Design, Setting, and Participants:**

This cohort study used data from the Cancer Prevention Study-II Nutrition Cohort and included men diagnosed with nonmetastatic prostate cancer between 1992 and 2003 and followed up from postdiagnosis survey completion until death or December 31, 2020. Analyses were conducted from March 2024 to February 2025.

**Exposure:**

Prediagnosis and postdiagnosis ACS guideline concordance was scored from 0 to 8, with higher scores indicating greater adherence.

**Main Outcomes and Measures:**

Mortality outcomes, including all-cause mortality (ACM), CVDM, and PCSM, were ascertained via National Death Index linkage. Cox proportional hazards regressions were used to estimate multivariable-adjusted hazard ratios (HRs) and 95% CIs across score categories (categorized as 0-3, 4, 5, and 6-8).

**Results:**

Among 4232 prostate cancer survivors (median [IQR] age at diagnosis, 69 [65.0-73.0] years) followed for a median (IQR) of 14.1 (8.4-17.5) years, there were 3101 ACM outcomes (73.3%), 912 CVDM outcomes (21.6%), and 453 PCSM outcomes (10.7%). Higher postdiagnosis ACS score (ie, 6 to 8 vs 0 to 3) was associated with lower risk of ACM (HR, 0.77; 95% CI, 0.69-0.85; per 1-point increase: HR, 0.94; 95% CI, 0.91-0.96; *P* < .001) and CVDM (HR, 0.75; 95% CI, 0.63-0.91; per 1-point increase: HR, 0.93; 95% CI, 0.89-0.97; *P* = .001), while results for PCSM were not statistically significant. Results were similar when stratified by tumor aggressiveness. Patients who improved their guideline concordance after diagnosis (ie, a score of <5 to ≥5) or maintained high concordance (ie, a score of ≥5 to ≥5) had lower mortality risk compared with those who maintained low scores.

**Conclusions and Relevance:**

In this cohort study of men with nonmetastatic prostate cancer, higher postdiagnosis ACS guideline concordance and improvements in concordance were associated with lower ACM and CVDM, highlighting the importance of lifestyle modifications in prostate cancer survivorship.

## Key Points

**Question:**

Is adherence to the American Cancer Society (ACS) Nutrition and Physical Activity Guideline for Cancer Survivors after a prostate cancer diagnosis associated with lower mortality risk?

**Findings:**

In this cohort study of 4232 men with nonmetastatic prostate cancer, higher ACS guideline concordance after diagnosis was associated with a 23% lower risk of all-cause mortality and a 25% lower risk of cardiovascular disease mortality; both results were statistically significant. Patients who improved their concordance after diagnosis experienced meaningful reductions in mortality risks.

**Meaning:**

These findings suggest that following the ACS guideline may reduce risk of mortality in men with nonmetastatic prostate cancer, highlighting the importance of lifestyle interventions during survivorship care.

## Teaser

This cohort study examines the association between patient adherence to postdiagnosis American Cancer Society guidelines for nutrition and physical activity and mortality among survivors of nonmetastatic prostate cancer.

## Introduction

Prostate cancer is the most commonly diagnosed cancer among men in the US in 2024, accounting for 29% of all male cancer diagnoses.^1^ Among men with nonmetastatic prostate cancer, prostate cancer–specific mortality (PCSM) is the leading cause of cancer-related death, while cardiovascular disease mortality (CVDM) is the most common non–cancer-related cause.^2^ Some studies suggest that PCSM^3^ and CVDM^4^ may be preventable through healthy lifestyle behaviors, underscoring the need to investigate modifiable factors associated with survival in this population.

The 2022 American Cancer Society (ACS) Nutrition and Physical Activity Guideline for Cancer Survivors recommends avoiding obesity, engaging in regular physical activity, and following a healthy diet.^5^ It also advises adherence to the ACS Guideline for Diet and Physical Activity for Cancer Prevention, which supports the above and recommends limiting alcohol intake.^5,6^ While individual guideline components have been associated with survival in men with prostate cancer,^7,8,9^ few studies have examined their combined effect. Previous studies have been limited by short follow-up periods,^10^ small sample sizes,^11^ and a primary focus on PCSM,^10,11,12^ despite CVDM being the leading competing cause of death among patients with nonmetastatic prostate cancer.^13^ Moreover, the impact of postdiagnosis lifestyle changes on survival remains unclear.

To address these gaps, we evaluated the association between postdiagnosis concordance with the ACS guideline and all-cause mortality (ACM), CVDM, and PCSM among patients with nonmetastatic prostate cancer in the Cancer Prevention Study-II Nutrition Cohort. We further examined whether these associations varied by tumor aggressiveness^14^ and assessed outcomes associated with changes in guideline concordance from prediagnosis to postdiagnosis.

## Methods

### Study Population

The Cancer Prevention Study-II (CPS-II) Nutrition Cohort, a subcohort of 1.2 million participants in the 1982 CPS-II mortality cohort,^15^ is a prospective study of 86 404 men and 97 786 women, established in 1992 to investigate the association between diet, lifestyle, and other factors and cancer incidence and mortality.^16^ Self-reported education, race, and date of birth data were collected on the 1982 baseline survey of the CPS-II.^16^ Race and ethnicity were self-reported at enrollment using separate questions for Hispanic or Latino ethnicity and race, with options: American Indian or Alaskan Native, Asian, Black or African American, Native Hawaiian or Pacific Islander, White, and other (with a write-in option); multiple selections were allowed. Race and ethnicity were assessed to describe the study population. Medical history, physical activity, smoking, diet, and other lifestyle factors were collected through questionnaires in the 1992 to 1993 baseline survey of the CPS-II Nutrition Cohort. Biennial follow-up surveys updated exposure data and identified new cancer diagnoses from 1997 to 2017.^16,17^ At the time of each mailed survey, participants were informed that their identifying information would be used to link with cancer registries and death indexes. Participants provided written informed consent for obtaining medical records, and all aspects of the study were approved by Emory University institutional review board. This cohort study followed the Strengthening the Reporting of Observational Studies in Epidemiology (STROBE) reporting guideline.

We identified 7339 men with first primary incident prostate cancer diagnosed between 1992 and 2003 (the year of the last diet assessment). We excluded 234 patients (3.2%) whose diagnoses were verified only by death certificate, 265 (3.6%) with missing tumor stage, 24 (0.3%) with unknown diagnosis date, and 163 (2.2%) with distant-stage tumors. We also excluded 389 men (5.3%) without a postdiagnosis survey, 792 men (10.8%) who returned the survey within 1 year of diagnosis, and 1065 men (14.5%) with missing ACS guideline score components (Figure). To minimize reverse causation and confounding from underlying health conditions, 35 men (0.5%) with a body mass index (BMI; calculated as weight in kilograms divided by height in meters squared) of less than 18.5 were excluded.^18^ Also, patients who are underweight may have distinct nutritional needs that may not be fully addressed by adherence to the ACS guideline, which is designed for the broader cancer patient population. Given tobacco’s strong impact on cancer and mortality outcomes,^19^ and because the Guideline on Nutrition and Physical Activity emphasizes behaviors apart from smoking, 140 men (1.9%) who currently smoked were also excluded.^5^ The final analytic cohort included 4232 prostate cancer survivors (57.7%) (Figure). Excluded participants had similar demographic characteristics but were slightly older (median [IQR] age, 71.0 [67.0-76.0] vs. 69.0 [65.0-73.0] years) and were more likely to have missing data on physical activity and dietary adherence measures.

**Figure.**
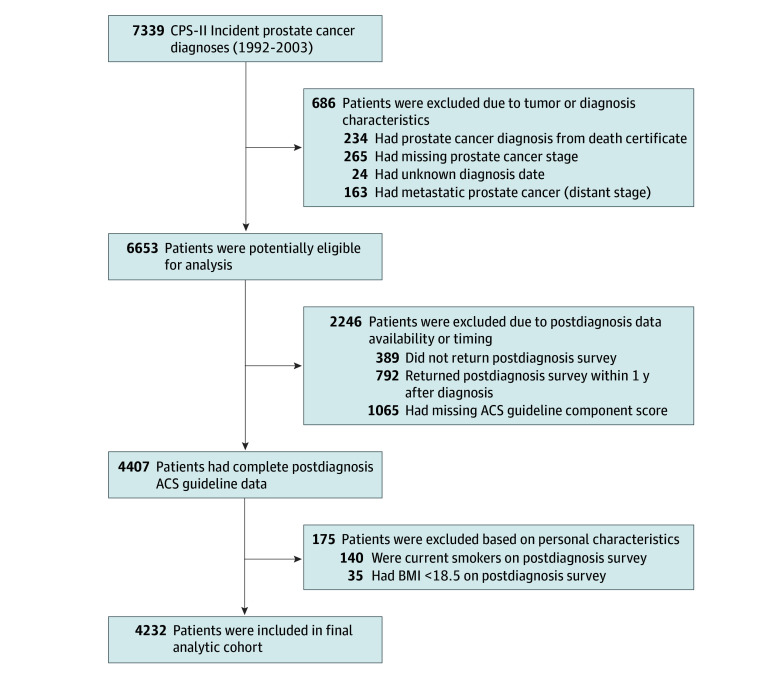
Study Flowchart of Exclusions to Identify Eligible Men Diagnosed With Prostate Cancer, 1992-2003 ACS indicates American Cancer Society; BMI, body mass index (calculated as weight in kilograms divided by height in meters squared); CPS-II, Cancer Prevention Study-II.

### ACS Guideline Score Assessment

Concordance with the ACS guideline was quantified according to the 2022 ACS guidelines for cancer survivors for body weight, physical activity, diet, and alcohol intake.^5^ The ACS guideline score was originally developed to assess concordance with the 2006 ACS Nutrition and Physical Activity Guidelines for Cancer Prevention.^17^ We adapted this score to align it with the 2022 ACS Nutrition and Physical Activity Guideline for Cancer Survivors.^5^ Details for scoring are provided in eMethods, eTable 1, and eTable 2 in Supplement 1.^17^ Briefly, each guideline component was scored from 0 to 2, with 2 points indicating optimal concordance with the guideline. The component scores were summed, resulting in a total ACS guideline score ranging from 0 to 8, with higher scores indicating higher concordance. The postdiagnosis guideline score was derived from the 1999 or 2003 follow-up survey, whichever was completed first and at least 1 year after diagnosis. The prediagnosis guideline score was calculated using the 1992 to 1993 baseline survey.

### Outcome Assessment

The primary outcome was ACM, and secondary outcomes were CVDM and PCSM. Causes of death were determined via linkage with the National Death Index, with CVDM (*International Statistical Classification of Diseases and Related Health Problems, Tenth Revision* [*ICD-10*] I00-I99) identified as the primary cause of death and PCSM (*ICD-10* C61) identified as the primary cause of death or as any cause of death if the primary cause of death was a cancer other than prostate cancer. Follow-up began at postdiagnosis survey completion using a delayed-entry model and ended at death or December 31, 2020, whichever occurred first.

### Statistical Analyses

Cox proportional hazards regression models estimated hazard ratios (HRs) and 95% CIs for ACM, CVDM, and CSM across ACS guideline score categories (analyzed continuously; categorized as 0 to 3, 4, 5, and 6 to 8) and its 4 sub-components (analyzed continuously; for BMI categorized as 0 or 2; and for other components scores categorized as 0, 1, or 2). This categorization of guideline scores was chosen to capture meaningful differences in concordance levels, as scores of 6 to 8 reflected concordance with most ACS recommendations, while scores of 0 to 3 reflected minimal concordance. For cause-specific mortality analyses, competing events were censored at the time of occurrence. Models were stratified by single year of age at diagnosis and adjusted for race and ethnicity, education, postdiagnosis smoking status and years since quitting, diabetes status, CVD, year of diagnosis, surgical, chemotherapy, and radiation treatment (Table 1). When individual ACS guideline components were analyzed separately, models were mutually adjusted for the other guideline components. Analyses were stratified by tumor aggressiveness, defined as nonaggressive (Gleason score 2 to 7 and tumor stage 1 to 2) or aggressive (Gleason score 8 to 10 or tumor stage 3 to 4) according to the National Comprehensive Cancer Network Guidelines.^14^

**Table 1.** Postdiagnosis Clinical, Demographic, and Lifestyle Characteristics by American Cancer Society Guideline Score Among 4232 Men Diagnosed With Nonmetastatic Prostate Cancer in the Cancer Prevention Study-II Nutrition Cohort (1992-2003)

Characteristic	Total (N = 4232)	ACS Guideline Score, No. (%)^a^			
		0-3 (n = 818)	4 (n = 802)	5 (n = 990)	6-8 (n = 1622)
Age at diagnosis, median (IQR), y	69 (65-73)	68 (65-72)	69 (65-73)	69 (66-73)	69 (66-73)
Age at diagnosis, y					
<65	848 (20)	201 (24.6)	165 (20.6)	190 (19.2)	292 (18.0)
65 to <70	1426 (33.7)	293 (35.8)	241 (30.0)	344 (34.7)	548 (33.8)
70 to <75	1246 (29.4)	213 (26.0)	258 (32.2)	285 (28.8)	490 (30.2)
75 to <80	601 (14.2)	94 (11.5)	118 (14.7)	146 (14.7)	243 (15.0)
≥80	111 (2.6)	17 (2.1)	20 (2.5)	25 (2.5)	49 (3.0)
Race and ethnicity^b^^,^^c^					
Black and Black Hispanic	46 (1.1)	9 (1.1)	10 (1.2)	14 (1.4)	13 (0.8)
White and White Hispanic	4143 (97.9)	801 (97.9)	782 (97.5)	968 (97.8)	1592 (98.2)
Other or unknown	43 (1.0)	8 (1.0)	10 (1.2)	8 (0.8)	17 (1.0)
Education^c^					
Less than high school	231 (5.5)	59 (7.2)	49 (6.1)	52 (5.3)	71 (4.4)
High school graduate	650 (15.4)	155 (18.9)	140 (17.5)	151 (15.3)	204 (12.6)
Some college	1013 (23.9)	210 (25.7)	198 (24.7)	248 (25.1)	357 (22.0)
College graduate	2338 (55.2)	394 (48.2)	415 (51.7)	539 (54.4)	990 (61.0)
Smoking status and years since quitting^d^					
Never smoked	1552 (36.7)	224 (27.4)	266 (33.2)	369 (37.3)	693 (42.7)
Quit <10 y ago	206 (4.9)	64 (7.8)	49 (6.1)	45 (4.5)	48 (3.0)
Quit 10 to <20 y ago	381 (9.0)	117 (14.3)	90 (11.2)	78 (7.9)	96 (5.9)
Quit ≥20 y ago	2048 (48.4)	402 (49.1)	391 (48.8)	488 (49.3)	767 (47.3)
Quit unknown y ago	45 (1.1)	11 (1.3)	6 (0.7)	10 (1.0)	18 (1.1)
Diagnosis year					
1992-1995	1205 (28.5)	255 (31.2)	220 (27.4)	275 (27.8)	455 (28.1)
1996-1999	1944 (45.9)	382 (46.7)	363 (45.3)	462 (46.7)	737 (45.4)
2000-2003	1083 (25.6)	181 (22.1)	219 (27.3)	253 (25.6)	430 (26.5)
Tumor stage					
T1-T2	3845 (90.9)	730 (89.2)	741 (92.4)	898 (90.7)	1476 (91.0)
T3-T4	387 (9.1)	88 (10.8)	61 (7.6)	92 (9.3)	146 (9.0)
Nodal involvement					
No	4185 (98.9)	806 (98.5)	791 (98.6)	981 (99.1)	1607 (99.1)
Yes	47 (1.1)	12 (1.5)	11 (1.4)	9 (0.9)	15 (0.9)
Gleason score					
2-6	2393 (56.5)	450 (55.0)	444 (55.4)	568 (57.4)	931 (57.4)
7	919 (21.7)	187 (22.9)	183 (22.8)	199 (20.1)	350 (21.6)
8-10	438 (10.3)	85 (10.4)	81 (10.1)	110 (11.1)	162 (10.0)
Unknown	482 (11.4)	96 (11.7)	94 (11.7)	113 (11.4)	179 (11.0)
NCCN tumor aggressiveness^e^					
Nonaggressive	3379 (79.8)	642 (78.5)	640 (79.8)	792 (80.0)	1305 (80.5)
Aggressive	749 (17.7)	155 (18.9)	136 (17.0)	179 (18.1)	279 (17.2)
Unknown	104 (2.5)	21 (2.6)	26 (3.2)	19 (1.9)	38 (2.3)
Surgery treatment					
No	1879 (44.4)	359 (43.9)	387 (48.3)	434 (43.8)	699 (43.1)
Yes	1952 (46.1)	373 (45.6)	350 (43.6)	443 (44.7)	786 (48.5)
Unknown	401 (9.5)	86 (10.5)	65 (8.1)	113 (11.4)	137 (8.4)
Chemotherapy					
No	3771 (89.1)	715 (87.4)	726 (90.5)	865 (87.4)	1465 (90.3)
Yes	60 (1.4)	17 (2.1)	11 (1.4)	12 (1.2)	20 (1.2)
Unknown	401 (9.5)	86 (10.5)	65 (8.1)	113 (11.4)	137 (8.4)
Radiotherapy					
No	2074 (49.0)	386 (47.2)	357 (44.5)	475 (48.0)	856 (52.8)
Yes	1757 (41.5)	346 (42.3)	380 (47.4)	402 (40.6)	629 (38.8)
Unknown	401 (9.5)	86 (10.5)	65 (8.1)	113 (11.4)	137 (8.4)
Diabetes^d^					
No	3622 (85.6)	670 (81.9)	661 (82.4)	865 (87.4)	1426 (87.9)
Yes	610 (14.4)	148 (18.1)	141 (17.6)	125 (12.6)	196 (12.1)
CVD^d^					
No	2926 (69.1)	547 (66.9)	529 (66.0)	699 (70.6)	1151 (71.0)
Yes	1306 (30.9)	271 (33.1)	273 (34.0)	291 (29.4)	471 (29.0)
BMI score^d^					
0 (≥30)	627 (14.8)	435 (53.2)	118 (14.7)	63 (6.4)	11 (0.7)
2 (18.5 to <30)	3605 (85.2)	383 (46.8)	684 (85.3)	927 (93.6)	1611 (99.3)
BMI, median (IQR)^d^	25.9 (24-28.5)	30.3 (26.3-32.1)	26.4 (24.4-28.6)	25.8 (23.8-27.8)	25.1 (23.4-27)
Physical activity score^d^					
0 (<7.5 MET h/wk)	1396 (33.0)	593 (72.5)	412 (51.4)	306 (30.9)	85 (5.2)
1 (7.5-15 MET h/wk)	996 (23.5)	155 (18.9)	248 (30.9)	277 (28.0)	316 (19.5)
2 (>15 MET h/wk)	1840 (43.5)	70 (8.6)	142 (17.7)	407 (41.1)	1221 (75.3)
Physical activity, median (IQR), MET h/wk^d^	14 (7-24.5)	7 (3.5-8.5)	7 (4.4-14)	14 (7-24.5)	24.5 (15.1-37)
Diet score^d^^,^^f^					
0 (lowest tertile; <5.25)	1354 (32.0)	582 (71.1)	373 (46.5)	320 (32.3)	79 (4.9)
1 (5.25 to <7.25)	1434 (33.9)	193 (23.6)	346 (43.1)	381 (38.5)	514 (31.7)
2 (highest tertile; ≥7.25)	1444 (34.1)	43 (5.3)	83 (10.3)	289 (29.2)	1029 (63.4)
Alcohol score^d^					
0 (>2 drinks/d)	583 (13.8)	211 (25.8)	165 (20.6)	115 (11.6)	92 (5.7)
1 (≤2 drinks/d)	2771 (65.5)	537 (65.6)	478 (59.6)	704 (71.1)	1052 (64.9)
2 (0 drink/d)	878 (20.7)	70 (8.6)	159 (19.8)	171 (17.3)	478 (29.5)
Alcohol intake, median (IQR), drinks/d^d^	0.3 (0-1.1)	0.5 (0.1-2.1)	0.3 (0-1.4)	0.3 (0-1.1)	0.2 (0-1)

Abbreviations: ACS guideline, American Cancer Society nutrition and physical activity guideline for cancer survivors; BMI, body mass index (calculated as weight in kilograms divided by height in meters squared); CPS-II, Cancer Prevention Study-II; CVD, cardiovascular disease; MET h/wk, metabolic equivalent of task h/wk; NCCN, National Comprehensive Cancer Network.

^a^ Data are presented as number (percentage) of patients unless otherwise indicated. Postdiagnosis characteristics were assessed in 1999 or 2003 follow-up surveys, whichever was completed first and at least 1 year after diagnosis.

^b^ Other/unknown category included participants who identified as American Indian or Alaskan Native, Asian, Native Hawaiian or Pacific Islander, more than 1 race, another race not listed, or did not report race or ethnicity in the 1982 CPS-II survey.

^c^ Assessed in prediagnosis 1982 CPS-II survey.

^d^ Assessed from postdiagnosis survey.

^e^ Tumor aggressiveness was defined by combining Gleason score and T-stage: nonaggressive tumors had a Gleason score of 2-7 and tumor stage T1-T2, while aggressive tumors had a Gleason score of 8-10 or tumor stage T3-T4.

^f^ Diet score (range: 0-12) was developed based on intakes of fruits and vegetables, a variety of fruits and vegetables, whole grains, red/processed meat, highly processed food or refined grain, and sugar-sweetened beverages. Higher scores indicate higher concordance with ACS guideline for diet.

To evaluate the impact of changes in the ACS guideline concordance after diagnosis, we classified participants into 4 groups: consistently low concordance (score less than 5 prediagnosis and less than 5 postdiagnosis), improved concordance (less than 5 prediagnosis and 5 or more postdiagnosis), decreased concordance (5 or more prediagnosis and less than 5 postdiagnosis), and consistently high concordance (more than 5 prediagnosis and more than 5 postdiagnosis). The consistently low concordance group served as the reference.

We conducted 2 stratified analyses by postdiagnosis BMI^9^ and CVD^20^ status, respectively. For analysis stratified by BMI, we used alternative ACS guideline score excluding the BMI (score ranging from 0 to 6 were categorized as 0 to 2, 3, 4, and 5 to 6) with a BMI of 25 as the cut-off point chosen to ensure similar subgroup sizes for better comparability.

Several sensitivity analyses were also conducted (1) using Fine-Gray models to calculate subdistribution hazard ratios (sdHR) and assess the effect of competing events on CVDM using Fine–Gray models to calculate subdistribution hazard ratios (sdHR) for CVDM and PCSM, accounting for other causes of death as competing events.^21^; (2) excluding individuals who formerly smoked and quit within 10 years of the primary diagnosis to assess the confounding by smoking history^22,23,24^; and (3) excluding deaths within 2 years of follow-up to address reverse causality bias.^24,25,26^ Data without missingness were used for primary analyses to maintain consistency with prior studies and simplify interpretation. To address potential selection bias due to conditioning on patients with prostate cancer who had nonmissing score,^27,28,29,30^ we applied multiple imputations (eFigure 1 in Supplement 1). Details of sensitivity analyses results are provided in the eMethods in Supplement 1.

A 2-sided *P* < .05 was considered statistically significant and an indicator of potential association. All statistical analyses were conducted using SAS 9.4 (SAS Institute) between March 2024 and February 2025.

## Results

### Participant Characteristics

Among 4232 prostate cancer survivors, the median (IQR) age at diagnosis was 69 (65-73) years (Table 1). A total of 1944 patients (45.9%) were diagnosed between 1996 and 1999, and 3379 (79.8%) had nonaggressive tumors. Forty-six participants (1.1%) were Black or Black Hispanic, and 4143 (97.9%) were White or White Hispanic. Compared with those with the lowest scores (ie, 0 to 3), patients with the highest postdiagnosis ACS guideline scores (ie, 6 to 8) were more likely to have a lower BMI, have never smoked, hold a college degree, and engage in moderate-to-vigorous physical activity (MVPA) exceeding 15 metabolic equivalent of task hours per week (MET hrs/wk).

Over a median (IQR) follow-up of 14.1 (8.4-17.5) years, there were 3101 (73.3%) ACM, 912 (21.6%) CVDM, and 453 (10.7%) PCSM outcomes. Median (IQR) time between prediagnosis survey completion and diagnosis was 4.8 (2.6-7.0) years and 3.4 (2.2-4.6) years between diagnosis and postdiagnosis survey completion. eFigure 2 in Supplement 1 shows the cumulative incidence of ACM, CVDM, and PCSM.

### Total Postdiagnosis ACS Guideline Score

Among all patients with prostate cancer, a higher ACS guideline score (ie, 6 to 8) was associated with lower risk of ACM compared with a lower ACS guideline score (ie, 0 to 3) (HR, 0.77; 95% CI, 0.69-0.85; per 1-point increase: HR, 0.94; 95% CI, 0.91-0.96; *P* < .001) and CVDM (HR, 0.75; 95% CI, 0.63-0.91; per 1-point increase: HR, 0.93; 95% CI, 0.89-0.97; *P* = .001). Higher scores were associated with lower risk of PCSM per 1-point increase (HR, 0.93; 95% CI, 0.87-0.99; *P* = .02) but not overall (HR, 0.79; 95% CI, 0.60-1.03) (Table 2).

**Table 2.** Cox Proportional Hazard Ratios and 95% CIs for the Association of Postdiagnosis Total American Cancer Society (ACS) Guideline Score and Mortality Among 4232 Patients Diagnosed With Nonmetastatic Prostate Cancer Stratified by Prostate Tumor Aggressiveness in the Cancer Prevention Study-II Nutrition Cohort (1992-2020)

ACS Guideline Score	Patients, No.	ACM				CVDM				PCSM			
		Deaths	Person years	HR (95% CI)^a^	*P* value^b^	Deaths	Person years	HR (95% CI)^a^	*P* value^b^	Deaths	Person years	HR (95% CI)^a^	*P* value^b^
**All patients with prostate cancer**													
0-3	818	626	10 147	1 [Reference]	NA	182	10 147	1 [Reference]	NA	96	10 147	1 [Reference]	NA
4	802	595	10 274	0.87 (0.78-0.98)	NA	174	10 274	0.83 (0.67-1.02)	NA	90	10 274	0.96 (0.71-1.29)	NA
5	990	731	12 970	0.87 (0.78-0.97)	NA	203	12 970	0.79 (0.64-0.97)	NA	112	12 970	0.98 (0.73-1.30)	NA
6-8	1622	1149	22 236	0.77 (0.69-0.85)	NA	353	22 236	0.75 (0.63-0.91)	NA	155	22 236	0.79 (0.60-1.03)	NA
Per 1-point increase	NA	NA	NA	0.94 (0.91-0.96)	.001	NA	NA	0.93 (0.89-0.97)	.001	NA	NA	0.93 (0.87-0.99)	.02
**Patients with non-aggressive tumor^c^**													
0-3	642	484	8153	1 [Reference]	NA	146	8153	1 [Reference]	NA	59	8153	1 [Reference]	NA
4	640	470	8346	0.89 (0.78-1.01)	NA	142	8346	0.84 (0.66-1.06)	NA	53	8346	0.86 (0.59-1.26)	NA
5	792	577	10 543	0.87 (0.77-0.99)	NA	165	10 543	0.82 (0.65-1.03)	NA	72	10 543	0.94 (0.66-1.35)	NA
6-8	1305	906	18 128	0.78 (0.70-0.87)	NA	290	18 128	0.79 (0.65-0.98)	NA	102	18 128	0.79 (0.57-1.11)	NA
Per 1-point increase	NA	NA	NA	0.94 (0.91-0.96)	<.001	NA	NA	0.95 (0.90-1.00)	.03	NA	NA	0.93 (0.86-1.01)	.07
**Patients with aggressive tumors^d^**													
0-3	155	123	1748	1 [Reference]	NA	30	1748	1 [Reference]	NA	32	1748	1 [Reference]	NA
4	136	102	1619	0.83 (0.62-1.09)	NA	24	1619	0.72 (0.40-1.29)	NA	33	1619	0.96 (0.57-1.62)	NA
5	179	137	2182	0.92 (0.70-1.20)	NA	35	2182	1.02 (0.59-1.77)	NA	36	2182	1.06 (0.63-1.79)	NA
6-8	279	213	3574	0.75 (0.58-0.95)	NA	53	3574	0.66 (0.40-1.10)	NA	48	3574	0.71 (0.43-1.16)	NA
Per 1-point increase	NA	NA	NA	0.94 (0.89-1.00)	.03	NA	NA	0.91 (0.81-1.01)	.08	NA	NA	0.92 (0.82-1.02)	.12

Abbreviations: ACM, all-cause mortality; CVDM, cardiovascular disease-related mortality; NA, not available; PCSM, prostate cancer–specific mortality.

^a^ Multivariable models were stratified by single year of age at diagnosis and adjusted for race, education, postdiagnosis smoking status and years since quitting, postdiagnosis diabetes history, postdiagnosis cardiovascular disease history, diagnosis year, surgery, chemotherapy, and radiation therapy. Overall prostate cancer models additionally adjusted for tumor stage, nodal involvement, and Gleason score.

^b^ *P* value for continuous ACS score variable.

^c^ Defined as tumor stage 1 to 2 and a Gleason score or 7 or less.

^d^ Defined as tumor stage 3 to 4 or a Gleason score more than 8 or nodal involvement.

When stratified by tumor aggressiveness, the results were largely the same but attenuated due to smaller sample sizes in subgroups. Among patients with nonaggressive tumors, higher score was associated with lower risk of ACM (HR, 0.78; 95% CI, 0.70-0.87; per 1-point increase: HR, 0.94; 95% CI, 0.91-0.96; *P*  < .001) and CVDM (HR, 0.79; 95% CI, 0.65-0.98; per 1-point increase: HR, 0.95; 95% CI, 0.90-1.00; *P* = .03), but not with PCSM (HR, 0.79; 95% CI, 0.57-1.11; per 1-point increase: HR, 0.93; 95% CI, 0.86-1.01; *P*  = .07). Similar patterns were observed in patients with aggressive tumors, for ACM (HR, 0.75; 95% CI, 0.58-0.95; per 1-point increase: HR, 0.94; 95% CI, 0.89-1.00; *P*  = .03), but not with CVDM (HR, 0.66; 95% CI, 0.40-1.10; per 1-point increase: HR, 0.91; 95% CI, 0.81-1.01; *P* = .08) or PCSM (HR, 0.71; 95% CI, 0.43-1.16; per 1-point increase: HR, 0.92; 95% CI, 0.82-1.02; *P*  = .12).

More details on the association between individual guideline components and mortality outcomes can be found in the eAppendix in Supplement 1 and Table 3. Overall, a postdiagnosis BMI of 18.5 to less than 30 and engaging in more than 15 MET h/wk of MVPA were associated with lower risks of ACM and CVDM, with MVPA also associated with a 33% lower risk of PCSM. In contrast, a higher diet score showed only a modest association with ACM risk, and alcohol abstinence was not significantly associated with mortality outcomes. Results were consistent for patients with both tumor types but less statistically significant for patients with aggressive tumors, which was likely due to the small sample size (eTable 3 in Supplement 1).

**Table 3.** Cox Proportional Hazard Ratios (HRs) and 95% CIs for the Association of Postdiagnosis Individual American Cancer Society Guideline Component Scores and Mortality Among 4232 Patients Diagnosed With Nonmetastatic Prostate Cancer in the Cancer Prevention Study-II Nutrition Cohort (1992-2020)

ACS Guideline Component, score	No.	ACM		*P* value^d^	CVDM		*P* value^d^	PCSM		*P* value^d^
		Deaths/person years	HR (95% CI)^a^		Deaths/person years	HR (95% CI)^a^		Deaths/ person years	HR (95% CI)^a^	
BMI^b^										
0	627	473/8026	1 [Reference]	NA	136/8026	1 [Reference]	NA	77/8026	1 [Reference]	NA
2	3605	2628/47 600	0.84 (0.76-0.94)	NA	776/47 600	0.81 (0.67-0.98)	NA	376/47 600	0.84 (0.65-1.09)	NA
Physical activity^c^										
0	1396	1104/16 434	1 [Reference]	NA	331/16 434	1 [Reference]	NA	179/16 434	1 [Reference]	NA
1	996	721/13 331	0.82 (0.74-0.90)	NA	217/13 331	0.82 (0.69-0.98)	NA	103/13 331	0.74 (0.58-0.96)	NA
2	1840	1276/25 862	0.80 (0.74-0.87)	NA	364/25 862	0.77 (0.66-0.90)	NA	171/25 862	0.67 (0.54-0.84)	NA
Per 1-point increase	NA	NA	0.90 (0.86-0.94)	<.001	NA	0.88 (0.81-0.95)	.001	NA	0.82 (0.73-0.92)	<.001
Diet^e^										
0	1354	1022/17 441	1 [Reference]	NA	296/17 441	1 [Reference]	NA	132/17 441	1 [Reference]	NA
1	1434	1037/18 854	0.95 (0.87-1.04)	NA	303/18 854	0.95 (0.80-1.12)	NA	167/18 854	1.25 (0.99-1.58)	NA
2	1444	1042/19 332	0.92 (0.84-1.00)	NA	313/19 332	0.93 (0.79-1.10)	NA	154/19 332	1.18 (0.92-1.50)	NA
Per 1-point increase	NA	NA	0.96 (0.92-1.00)	.06	NA	0.96 (0.89-1.05)	.38	NA	1.08 (0.96-1.22)	.20
Alcohol^f^										
0	583	404/7837	1 [Reference]	NA	104/7837	1 [Reference]	NA	69/7837	1 [Reference]	NA
1	2771	1994/37 117	0.97 (0.87-1.08)	NA	589/37 117	1.07 (0.87-1.33)	NA	292/37 117	0.86 (0.66-1.13)	NA
2	878	703/10 672	1.08 (0.95-1.23)	NA	219/10 672	1.19 (0.93-1.53)	NA	92/10 672	0.78 (0.56-1.09)	NA
Per 1-point increase	NA	NA	1.05 (0.98-1.12)	.15	NA	1.10 (0.97-1.24)	.14	NA	0.89 (0.75-1.05)	.16

Abbreviations: ACM, all-cause mortality; ACS guideline, American Cancer Society nutrition and physical activity guideline for cancer survivors; BMI, body mass index; CVDM, cardiovascular disease-related mortality; NA, not applicable; PCSM, prostate cancer–specific mortality.

^a^ Multivariable models were stratified by single year of age at diagnosis and adjusted for race, education, postdiagnosis smoking status and years since quitting, postdiagnosis diabetes history, postdiagnosis cardiovascular disease history, diagnosis year, surgery, chemotherapy, and radiation therapy. Overall prostate cancer models additionally adjusted for tumor stage, nodal involvement, and Gleason score.

^b^ BMI component score of 2 was assigned for a BMI between 18.5 to less than 30, and 0 for a BMI of 30 or more.

^c^ Physical Activity component score of 2 was assigned for more than 15 MET h/wk, 1 for 7.5 to 15 MET h/wk, and 0 for less than 7.5 MET h/wk.

^d^ *P* value for trend analysis across the continuous ACS score variable.

^e^ Diet component score of 2 was assigned for the highest tertile (7.25 or more) of the ACS Diet Score, 1 for the middle tertile (5.25 to less than 7.25), and 0 for the lowest tertile (less than 5.25).

^f^ Alcohol component score of 2 was assigned for 0 drinks per day, 1 for 2 or fewer drinks per day, and 0 for more than 2 drinks per day.

### Changes in ACS Guideline Scores

Of 1463 individuals with a prediagnosis ACS score of less than 5, 892 men (61.0%) maintained a consistently low concordance postdiagnosis, while 571 (39.0%) improved to 5 or more. Among 2322 individuals with a prediagnosis ACS score of 5 or more, 1802 (77.6%) had a consistently high concordance postdiagnosis while 520 (22.4%) decreased to less than 5 postdiagnosis.

Compared with those who had consistently low concordance, those who maintained higher concordance had a 20% lower risk of ACM (HR, 0.80; 95% CI, 0.73-0.88), a 21% lower risk of CVDM (HR, 0.79; 95% CI, 0.66-0.94), and a 32% lower risk of PCSM (HR, 0.68; 95% CI, 0.53-0.87) (Table 4). Patients who improved concordance had a 17% lower risk of ACM (HR, 0.83; 95% CI, 0.73-0.94) and a 28% lower risk of CVDM (HR, 0.72; 95% CI, 0.56-0.92); risk reduction for PCSM was not statistically significant (HR, 0.84; 95% CI, 0.62-1.15). Interestingly, men with higher prediagnosis concordance that decreased postdiagnosis also had a 14% lower risk of ACM (HR, 0.86; 95% CI, 0.76-0.98) and a 39% lower risk of PCSM (HR, 0.61; 95% CI, 0.43-0.86) but not significantly lower risk of CVDM (HR, 0.91; 95% CI, 0.72-1.14).

**Table 4.** Cox Proportional Hazard Ratios (HRs) and 95% CIs for the Association of Change From Prediagnosis to Postdiagnosis in American Cancer Society Guideline Score and Mortality Among 3785 Patients Diagnosed With Nonmetastatic Prostate Cancer in the Cancer Prevention Study-II Nutrition Cohort, 1992-2020^a^

ACS Guideline Score Change	No.	ACM			CVDM			PCSM		
		Deaths	Person years	HR (95% CI)	Deaths	Person years	HR (95% CI)	Deaths	Person years	HR (95% CI)
Low to low (<5 to <5)	892	659	11 366	1 [Reference]	187	11 366	1 [Reference]	110	11 366	1 [Reference]
Low to high (<5 to ≥5)	571	403	7767	0.83 (0.73-0.94)	104	7767	0.72 (0.56-0.92)	65	7767	0.84 (0.62-1.15)
High to low (≥5 to <5)	520	394	6699	0.86 (0.76-0.98)	124	6699	0.91 (0.72-1.14)	50	6699	0.61 (0.43-0.86)
High to high (≥5 to ≥5)	1802	1290	24 469	0.80 (0.73-0.88)	382	24 469	0.79 (0.66-0.94)	175	24 469	0.68 (0.53-0.87)

Abbreviations: ACM, all-cause mortality; ACS guideline, American Cancer Society nutrition and physical activity guideline for cancer survivors; CPS-II, Cancer Prevention Study-II; CVD, cardiovascular disease; CVDM, cardiovascular disease-related mortality; PCSM, prostate cancer–specific mortality.

^a^ Multivariable models were stratified by single year of age at diagnosis and adjusted for race, education, postdiagnosis smoking status and years since quitting, postdiagnosis diabetes history, postdiagnosis cardiovascular disease history, diagnosis year, surgery, chemotherapy, and radiation therapy. Overall prostate cancer models additionally adjusted for tumor stage, nodal involvement, and Gleason score.

### Supplemental Analyses

More details on the results of the stratified and sensitivity analyses can be found in the eAppendix of Supplement 1. Higher adherence to an alternative ACS guideline score (excluding the BMI component) was associated with a lower risk of ACM, but not CVDM or PCSM, among patients with a BMI less than 25, and with lower risks of ACM, CVDM, and PCSM among patients with BMI of 25 or more (eTable 4 in Supplement 1). Among patients without postdiagnosis CVD, higher guideline scores were associated with lower ACM but not CVDM or PCSM, while for those with CVD, higher scores were linked to lower risks of ACM and CVDM but not PCSM (eTable 5 in Supplement 1).

Fine-Gray models supported the primary analyses with some attenuation (eTable 6 in Supplement 1). Sensitivity analyses excluding patients who quit smoking within 10 years of the primary diagnosis, removing deaths within the first 2 years after the primary diagnosis, or imputing missing scores were consistent with the main results (eTable 7, eTable 8, and eTable 9 in Supplement 1).

## Discussion

In this large cohort of nonmetastatic prostate cancer survivors, higher postdiagnosis ACS guideline concordance, particularly avoiding obesity and engaging in more than 15 MET h/wk of MVPA, was associated with lower risks of ACM, CVDM, and PCSM. Results were similar across tumor aggressiveness strata, although attenuated for aggressive tumors, likely due to smaller sample sizes. Maintaining high concordance both before and after diagnosis was associated with significantly lower mortality, including a 32% reduction in PCSM, compared with consistently low concordance. Improving concordance after diagnosis was also associated with lower ACM and CVDM, highlighting the benefits of adopting healthy behaviors after diagnosis.

Previous studies found that postdiagnosis concordance with lifestyle recommendations was associated with a lower risk of PCSM,^10,11^ but our study is among the first to assess its effect on ACM and CVDM. To our knowledge, no prior studies assessed lifestyle scores by tumor aggressiveness, limiting comparisons. However, our findings suggest stronger associations for men with nonaggressive tumors, aligning with prior studies about BMI, MVPA and mortality among patients with less aggressive tumors.^8,9,31^

Higher MVPA volume was associated with a lower risk of ACM, CVDM, and PCSM, with guideline-consistent activity (7.5 to 15 MET h/wk) benefiting patients with nonaggressive tumors, while more than 15 MET h/wk was needed to reduce mortality risk across all patients. A weekly volume of 7.5 to 15 MET hours is approximately equivalent to 150 to 300 minutes of moderate intensity or 75 to 150 minutes of vigorous intensity aerobic activity, or a combination of both. These findings align with prior studies^12,32,33^ suggesting that vigorous intensity activity may lower ACM risk in prostate cancer survivors. While this study focuses on the benefits of MVPA, evidence also supports resistance training as a beneficial intervention for cancer survivors.^33,34^

Additionally, avoiding obesity was associated with lower ACM and CVDM risk, consistent with previous studies,^9^ reinforcing the importance of weight management in survivorship. While higher diet scores were inversely associated with ACM and CVDM, the lack of association with PCSM may reflect screening bias, which we cannot assess in this study. Similar findings from the Health Professionals Follow-Up Study suggested that greater postdiagnosis adherence to a Mediterranean diet was linked to lower ACM but not PCSM.^35^ Although the ACS and Mediterranean dietary patterns both emphasize plant-based foods, the latter uniquely prioritizes the consumption of olive oil, fish, seafood, and nuts. Abstaining from alcohol showed no association with mortality outcomes, consistent with prior research in prostate cancer survivors.^36^

### Strengths and Limitations

This study is among the largest to examine ACS guideline concordance and cause-specific mortality among prostate cancer survivors. Several sensitivity analyses support the robustness of our findings, and the results of the Fine-Gray models suggest the importance of considering how competing risks might attenuate the estimated effects.^37^

Residual confounding cannot be ruled out despite adjustment for many covariates. Self-reported data may introduce measurement error, which is likely nondifferential.^38^ The exclusion of metastatic cases may limit generalizability, and we lacked data on progression during follow-up. Although differences between included and excluded participants were minimal, selection bias remains a possibility; however, results using multiple imputation for missing data were consistent with the primary analysis. Additionally, the study population was predominantly White and highly educated, potentially limiting the generalizability of the findings to more diverse groups. Due to a small number of Hispanic White participants, we combined them with the non-Hispanic White participants, which may be a limitation. Additionally, we lacked data on several social determinants of health^39^ (eg, neighborhood deprivation, access to care) that may influence adherence to lifestyle recommendations, which should be explored in future studies. Finally, we lacked a more granular breakdown of the Gleason score, which may be relevant given the evolving debate on the classification of lower Gleason scores in current clinical practice.^40^

## Conclusions

In this prospective cohort study of nonmetastatic prostate cancer survivors, higher postdiagnosis concordance with the ACS Nutrition and Physical Activity Guideline for Cancer Survivors was associated with lower risk of ACM, CVDM, and PCSM, supporting recommendations for avoiding obesity, engaging in regular physical activity, following a healthy eating pattern, and limiting alcohol intake to improve long-term survival. The consistent association between higher scores and lower ACM risk, regardless of tumor aggressiveness, suggested the benefit of a healthy lifestyle after cancer diagnosis. Notably, improved guideline concordance postdiagnosis was associated with lower ACM risk, highlighting the importance of integrating lifestyle interventions, such as physical activity, weight management programs, and diet counseling into survivorship care. Future research should evaluate the feasibility and effectiveness of these interventions for improving guideline adherence and reducing mortality risk.

## Notes

Supplement 1.
eMethods.
**eAppendix.** Additional results **eTable 1.** 2022 American Cancer Society (ACS) nutrition and physical activity guideline for cancer survivors. **eTable 2.** The American Cancer Society (ACS) Diet score (out of 12) quantifies concordance with the diet component of the ACS Nutrition and Physical Activity Guidelines for Cancer Prevention in the Cancer Prevention Study-II Nutrition Cohort. **eFigure 1.** Directed Acyclic Graph (DAG) illustrating a collider bias and mitigating it using multiple imputation **eFigure 2.** Cumulative incidence of all-cause, cardiovascular disease-related, and prostate cancer–specific mortality stratified by ACS Guideline score among 4232 prostate cancer patients in the CPS-II Nutrition Cohort (1992-2020) **eTable 3.** Cox Proportional hazard ratios (HRs) and 95% CIs for the Association of Postdiagnosis individual American Cancer Society (ACS) guideline component scores and mortality among 4232 patients diagnosed with prostate cancer stratified by prostate cancer aggressiveness in the Cancer Prevention Study-II Nutrition Cohort (1992-2020) **eTable 4.** Cox Proportional hazard ratios (HRs) and 95% CIs for the association of postdiagnosis alternative American Cancer Society (ACS) guideline score (without body mass index [BMI] component) and mortality among 4232 patients diagnosed with prostate cancer stratified by postdiagnosis BMI in the Cancer Prevention Study-II Nutrition Cohort (1992-2020) **eTable 5.** Cox Proportional hazard ratios (HRs) and 95% CIs for the association of postdiagnosis total American Cancer Society (ACS) guideline score and mortality among 4232 patients diagnosed with prostate cancer stratified by prevalent cardiovascular disease reported at postdiagnosis survey in the Cancer Prevention Study-II Nutrition Cohort (1992-2020) **eTable 6.** Subdistribution hazard ratios (sdHR) and 95% CI for the association of postdiagnosis total American Cancer Society (ACS) guideline score and CVDM and PCSM among 4232 patients diagnosed with prostate cancer in the Cancer Prevention Study-II Nutrition Cohort (1992-2020) **eTable 7.** Cox Proportional hazard ratios (HRs) and 95% CIs for the association of postdiagnosis total American Cancer Society (ACS) guideline score and mortality among 4026 patients diagnosed with prostate cancer, excluding those who quit smoking within 10 years of postdiagnosis questionnaire return in the Cancer Prevention Study-II Nutrition Cohort (1992-2020) **eTable 8.** Cox Proportional hazard ratios (HRs) and 95% CIs for the association of postdiagnosis total American Cancer Society (ACS) guideline score and mortality among 4077 patients diagnosed with prostate cancer, excluding outcome events that occurred within the first 2 years of follow-up to account for reverse causality in the Cancer Prevention Study-II Nutrition Cohort (1992-2020) **eTable 9.** Cox Proportional hazard ratios (HRs) and 95% CIs for the association of postdiagnosis total American Cancer Society (ACS) guideline score and mortality among 5297 patients diagnosed with prostate cancer where missing postdiagnosis ACS guideline score were imputed if missing in the Cancer Prevention Study-II Nutrition Cohort (1992-2020)
eReferences.

Supplement 2.
Data Sharing Statement
