# Supplementary material for: Adherence to American Cancer Society Guideline and Mortality in Men With Nonmetastatic Prostate Cancer
Source: JAMA Netw Open. 2025 Sep 26;8(9):e2533922. doi: 10.1001/jamanetworkopen.2025.33922 (PMC12475947; doi:10.1001/jamanetworkopen.2025.33922)
Supplement: Supplement 1. — eMethods. eAppendix. Additional results eTable 1. 2022 American Cancer Society (ACS) nutrition and physical activity guideline for cancer survivors. eTable 2. The American Cancer Society (ACS) Diet score (out of 12) quantifies concordance with the diet component of the ACS Nutrition and Physical Activity Guidelines for Cancer Prevention in the Cancer Prevention Study-II Nutrition Cohort. eFigure 1. Directed Acyclic Graph (DAG) illustrating a collider bias and mitigating it using multiple imputation eFigure 2. Cumulative incidence of all-cause, cardiovascular disease-related, and prostate cancer–specific mortality stratified by ACS Guideline score among 4232 prostate cancer patients in the CPS-II Nutrition Cohort (1992-2020) eTable 3. Cox Proportional hazard ratios (HRs) and 95% CIs for the Association of Postdiagnosis individual American Cancer Society (ACS) guideline component scores and mortality among 4232 patients diagnosed with prostate cancer stratified by prostate cancer aggressiveness in the Cancer Prevention Study-II Nutrition Cohort (1992-2020) eTable 4. Cox Proportional hazard ratios (HRs) and 95% CIs for the association of postdiagnosis alternative American Cancer Society (ACS) guideline score (without body mass index [BMI] component) and mortality among 4232 patients diagnosed with prostate cancer stratified by postdiagnosis BMI in the Cancer Prevention Study-II Nutrition Cohort (1992-2020) eTable 5. Cox Proportional hazard ratios (HRs) and 95% CIs for the association of postdiagnosis total American Cancer Society (ACS) guideline score and mortality among 4232 patients diagnosed with prostate cancer stratified by prevalent cardiovascular disease reported at postdiagnosis survey in the Cancer Prevention Study-II Nutrition Cohort (1992-2020) eTable 6. Subdistribution hazard ratios (sdHR) and 95% CI for the association of postdiagnosis total American Cancer Society (ACS) guideline score and CVDM and PCSM among 4232 patients diagnosed with prostate cancer i [file jamanetwopen-e2533922-s001.pdf]

Supplemental Online Content

Elahy V, Newton CC, McCullough ML, et al. Following American Cancer Society Guideline and mortality in men with nonmetastatic prostate cancer. *JAMA Netw Open*. 2025;8(9):e2533922. doi:10.1001/jamanetworkopen.2025.33922

eMethods.
eAppendix. Additional results
eTable 1. 2022 American Cancer Society (ACS) nutrition and physical activity guideline for cancer survivors.
eTable 2. The American Cancer Society (ACS) Diet score (out of 12) quantifies concordance with the diet component of the ACS Nutrition and Physical Activity Guidelines for Cancer Prevention in the Cancer Prevention Study-II Nutrition Cohort.
eFigure 1. Directed Acyclic Graph (DAG) illustrating a collider bias and mitigating it using multiple imputation
eFigure 2. Cumulative incidence of all-cause, cardiovascular disease-related, and prostate cancer-specific mortality stratified by ACS Guideline score among 4232 prostate cancer patients in the CPS-II Nutrition Cohort (1992-2020)
eTable 3. Cox Proportional hazard ratios (HRs) and 95% CIs for the Association of Postdiagnosis individual American Cancer Society (ACS) guideline component scores and mortality among 4,232 patients diagnosed with prostate cancer stratified by prostate cancer aggressiveness in the Cancer Prevention Study-II Nutrition Cohort (1992-2020)
eTable 4. Cox Proportional hazard ratios (HRs) and 95% CIs for the association of postdiagnosis alternative American Cancer Society (ACS) guideline score (without body mass index [BMI] component) and mortality among 4232 patients diagnosed with prostate cancer stratified by postdiagnosis BMI in the Cancer Prevention Study-II Nutrition Cohort (1992-2020)
eTable 5. Cox Proportional hazard ratios (HRs) and 95% CIs for the association of postdiagnosis total American Cancer Society (ACS) guideline score and mortality among 4232 patients diagnosed with prostate cancer stratified by prevalent cardiovascular disease reported at postdiagnosis survey in the Cancer Prevention Study-II Nutrition Cohort (1992-2020)
eTable 6. Subdistribution hazard ratios (sdHR) and 95% CI for the association of postdiagnosis total American Cancer Society (ACS) guideline score and CVDM and PCSM among 4232 patients diagnosed with prostate cancer in the Cancer Prevention Study-II Nutrition Cohort (1992-2020)
eTable 7. Cox Proportional hazard ratios (HRs) and 95% CIs for the association of postdiagnosis total American Cancer Society (ACS) guideline score and mortality among 4026 patients diagnosed with prostate cancer, excluding those who quit smoking within 10 years of postdiagnosis questionnaire return in the Cancer Prevention Study-II Nutrition Cohort (1992-2020)
eTable 8. Cox Proportional hazard ratios (HRs) and 95% CIs for the association of postdiagnosis total American Cancer Society (ACS) guideline score and mortality among 4077 patients diagnosed with prostate cancer, excluding outcome events that occurred within the first 2 years of follow-up to account for reverse causality in the Cancer Prevention Study-II Nutrition Cohort (1992-2020)
eTable 9. Cox Proportional hazard ratios (HRs) and 95% CIs for the association of postdiagnosis total American Cancer Society (ACS) guideline score and mortality among 5297 patients diagnosed with prostate cancer where missing postdiagnosis ACS guideline score were imputedb if missing in the Cancer Prevention Study-II Nutrition Cohort (1992-2020)
eReferences.

This supplemental material has been provided by the authors to give readers additional information about their work.

© 2025 Elahy V et al. *JAMA Network Open*.

eMethods
Covariate Data
Smoking status was derived from the postdiagnosis survey responses and categorized into 5 groups (never-smokers, former smokers quitting <10 years, 10-<20, ≥20, or an unknown number of years before the postdiagnosis questionnaire). Postdiagnosis diabetes status was identified by self-reported diabetes or self-reported use of insulin or oral diabetes medications, and cardiovascular disease (CVD) status was determined through a self-reported history of coronary artery disease, stroke, myocardial infarction, or transient ischemic attack. Missing data on diabetes and CVD were carried forward if previously reported. The first course of treatment was self-reported by patients on their postdiagnosis survey and was defined as surgery (yes, no, unknown), chemotherapy (yes, no, unknown), and radiation therapy (yes, no, unknown).

Prostate cancer tumor characteristics, including T-stage, N-stage, and Gleason score, were abstracted from clinical and pathological records. T-stage was categorized as '1-2' for localized tumors and '3-4' for advanced tumors, while N-stage was recorded as '0' for no lymph node involvement and '1' for involvement. Gleason scores were grouped into 1-4 (low), 5-6 (intermediate), 7 (moderate), and 8-10 (high-grade). Tumor aggressiveness was defined as non-aggressive (Gleason score 2-7 and T-stage 1-2) or aggressive (Gleason score 8-10 or T-stage 3-4).[1]

Individual Guideline Components
Concordance with the recommendation to avoid obesity was assessed using body mass index (BMI), which was calculated using self-reported height from the 1982 baseline survey and weight from pre- or postdiagnosis surveys.[2] A BMI of 18.5-<30 kg/m^2 (concordant with the definition of avoiding obesity) was assigned 2 points, and a BMI ≥30 kg/m^2 (obesity) was assigned 0 points.

Concordance with the recommendation to engage in regular physical activity was assessed using self-reported data on time spent on recreational moderate to vigorous-intensity aerobic physical activities (MVPA) such as walking, jogging, and swimming from the 1992, 1999, and 2001 Cancer Prevention Study-II (CPS-II) Nutrition Cohort surveys, with 2001 values carried forward to 2003 (due to survey changes in 2003).[3] MVPA was quantified in Metabolic Equivalent of Task (MET) hours per week (MET-hrs/wk), derived by multiplying the weekly minutes spent in each reported activity by its standard MET value. To account for the range of intensity reported by participants, activities were classified using the Compendium of Physical Activities as moderate (3.0–5.9 METs) or vigorous (≥6.0 METs).[4] A conservative approach was taken by assigning 3.0 METs for moderate and 6.0 METs for vigorous activities to reduce the risk of overestimating physical activity.[4] For example, 30 minutes/day of moderate-intensity activity across 5 days (2.5 hours/week) translates to 7.5 MET-hrs/wk. This method aligns with other studies in the CPS cohorts using self-reported MVPA to estimate MET-hrs/wk.[5] Concordance was scored based on ACS guideline thresholds: <7.5 MET-hrs/wk (0 points), 7.5–15 MET-hrs/wk (1 point), and >15 MET-hrs/wk (2 points), which corresponds to the general recommendation of at least 150 minutes/wk of moderate-intensity or 75 minutes/wk of vigorous-intensity physical activity.

The diet and alcohol components of the ACS Guideline score were evaluated using dietary data from the Block Food Frequency Questionnaire (FFQ) in 1992/1993[6] and the Willett FFQ in 1999 or 2003[7] (first returned ≥1 year postdiagnosis). Concordance with the recommendation to follow a healthy eating pattern was assessed based on the intake of fruits and vegetables, whole grains, red and processed meats, sugar-sweetened beverages, highly processed foods, and refined grains. The diet score ranges from 0-12 points (**eTable 2**), and was then categorized into tertiles, where participants in the highest tertile received 2 points, those in the second tertile 1-point, and those in the lowest tertile 0 points.[8] These points contributed to the total ACS Guideline score.

For alcohol intake, compliance was scored with 0 points for ≥2 drinks/day, 1-point for >0-<2 drinks/day, and 2 points for no alcohol intake.

Modeling Considerations
Although follow-up surveys were conducted biennially, complete data for all components of the ACS guideline score were only available at two time points: 1992/1993 (pre-diagnosis) and either 1999 or 2003 (postdiagnosis). Out of 4,232 eligible participants, only 1,654 (39.1%) participants had score components available at both 1999 and 2003, limiting our ability to treat ACS guideline concordance as a time-varying exposure. Using a time-varying exposure would have required restricting the analysis to this subgroup, leading to loss of power and potential

© 2025 Elahy V et al. *JAMA Network Open*.

selection bias. Therefore, we focused on a single postdiagnosis measurement to maximize generalizability and maintain a consistent analytic cohort.

eAppendix. Additional results

Individual Guideline Components

Compared with BMI ≥30.0 kg/m², BMI of 18.5 to <30.0 kg/m² was associated with lower risk of all-cause mortality (ACM; HR, 0.84; 95% CI, 0.76–0.94) and CVD mortality (CVDM; HR, 0.81; 95% CI, 0.67–0.98), but not prostate cancer–specific mortality (PCSM; HR, 0.84; 95% CI, 0.65–1.09) (**Table 3**).

For physical activity, engaging in moderate to vigorous-intensity recreational aerobic physical activity with intensity of >15 MET-hrs/wk was associated with a 20% lower risk of ACM (HR, 0.80; 95% CI, 0.74 to 0.87), a 23% lower risk of CVDM (HR, 0.77; 95% CI, 0.66 to 0.90), and a 33% lower risk of PCSM (HR, 0.67; 95% CI, 0.54 to 0.84) compared to physical activity<7.5 MET-hrs/wk.

A diet score in the highest tertile was associated with an 8% lower risk of ACM (HR, 0.92; 95% CI, 0.84 to 1.00), but not with CVDM (HR, 0.93; 95% CI, 0.79 to 1.10), or PCSM (HR, 1.18; 95% CI, 0.92 to 1.50), compared to the lowest tertile.

Consistent with another study reporting no significant association between alcohol intake and ACM or PCSM in prostate cancer survivors.[9]Abstaining from alcohol (0 drinks/day) was not significantly associated with ACM (HR, 1.08; 95% CI, 0.95 to 1.23), CVDM (HR, 1.19; 95% CI, 0.93 to 1.53), or PCSM (HR, 0.78; 95% CI, 0.56 to 1.09) compared to consuming ≥2 drinks/day.

Consistent with prior research,[10] we found that a higher volume of moderate-to-vigorous physical activity was associated with lower risks of ACM, CVDM, and PCSM, with guideline-consistent volume of physical activity (7.5-15 MET-hrs/week) benefiting patients with non-aggressive tumors, while higher volume (>15 MET-hrs/week) was required to lower mortality risk among all patients, regardless of tumor type. Based on 3,101 ACM and 453 PCSM outcomes, our findings are more robust than prior studies with smaller sample sizes and align with an emulated target trial suggesting vigorous activity may reduce ACM risk in prostate cancer survivors.[11] Although this study focuses on the benefits of moderate-to-vigorous activity for patients with prostate cancer, it is important to note the growing body of evidence supporting the role of resistance training as a key intervention for cancer survivors[12,13].

Aligned with our previous findings,[14] avoiding obesity was also significantly associated with a lower risk of mortality outcomes, as men with a BMI of 18.5-<30 kg/m² had lower ACM and CVDM risk than those with BMI ≥30 kg/m², reinforcing the importance of weight management in survivorship.

Concordance with diet recommendations was inversely but not statistically significantly associated with lower risk of ACM and CVDM, but not PCSM, possibly due to screening bias. A healthier diet may be associated with frequent screenings and earlier tumor detection; however, it cannot be evaluated in this study. This was consistent with the findings from the Health Professionals Follow-up Study, where greater postdiagnosis concordance with the Mediterranean diet was associated with a lower risk of ACM but not PCSM.[15] Although both the ACS and Mediterranean dietary patterns emphasize plant-based foods, the Mediterranean diet uniquely prioritizes olive oil, moderate fish, seafood, and nut intake.

Stratified Analyses

Among patients with a postdiagnosis BMI<25 kg/m², higher alternative ACS guideline score (without the BMI component) was associated with lower risk of ACM (score 5-6 vs. 0-2: HR, 0.80; 95% CI, 0.67 to 0.95; per 1-point increase: HR, 0.95; 95% CI: 0.90 to 0.99; P-continuous=0.02), but not with risk of CVDM (score 5-6 vs. 0-2: HR, 0.81; 95% CI, 0.58 to 1.14; per 1-point increase: HR, 0.97; 95% CI: 0.88 to 1.05; P-continuous=0.44) or PCSM (score 5-6 vs. 0-2: HR, 1.14; 95% CI, 0.67 to 1.93; per 1-point increase: HR, 1.01; 95% CI, 0.88 to 1.15; P-continuous=0.91) **(eTable 4)**. The highest score of 5-6 was not associated with mortality outcomes among patients with BMI ≥25 kg/m². Among patients with a postdiagnosis BMI ≥25 kg/m², higher alternative score (without the BMI component) was associated with a lower risk of ACM (score 5-6 vs. 0-2: HR, 0.89; 95% CI, 0.78 to 1.02; per 1-point increase: HR, 0.94; 95% CI: 0.91 to 0.97; P-continuous<0.001), CVDM (score 5-6 vs. 0-2: HR, 0.86; 95%

CI, 0.67 to 1.11; per 1-point increase: HR, 0.93; 95% CI: 0.88 to 1.00; P-continuous=0.03) and PCSM (score 5-6 vs. 0-2: HR, 0.87; 95% CI, 0.62 to 1.21; per 1-point increase: HR, 0.90; 95% CI, 0.83 to 0.98; P-continuous=0.02)

Among patients without postdiagnosis CVD, higher ACS guideline score was associated with lower risk of ACM (score 6-8 vs. 0-3: HR, 0.73; 95% CI, 0.64 to 0.83; per 1-point increase: HR, 0.93; 95% CI, 0.90 to 0.96; P-continuous<0.001), but not with risk of CVDM (score 6-8 vs. 0-3: HR, 0.78; 95% CI, 0.61 to 1.00; per 1-point increase: HR, 0.95; 95% CI, 0.90 to 1.01; P-continuous=0.11) or PCSM (score 6-8 vs. 0-3: HR, 0.73; 95% CI, 0.53 to 1.02; per 1-point increase: HR, 0.90; 95% CI, 0.84 to 0.97; P-continuous=0.005) **(eTable 5).** Among patients with CVD reported postdiagnosis, the highest score was associated with lower risk of ACM (score 6-8 vs. 0-3: HR, 0.82; 95% CI, 0.69 to 0.97; per 1-point increase: HR, 0.95; 95% CI, 0.91 to 0.98; P-continuous=0.005), CVDM (score 6-8 vs. 0-3: HR, 0.67; 95% CI, 0.50 to 0.91; per 1-point increase: HR, 0.90; 95% CI, 0.84 to 0.96; P-continuous=0.002), but not with risk of PCSM (score 6-8 vs. 0-3: HR, 0.89; 95% CI, 0.55 to 1.42; per 1-point increase: HR, 0.99; 95% CI, 0.89 to 1.10; P-continuous=0.87).

Competing Risk Analyses

For PCSM outcomes, deaths due to causes other than prostate cancer are considered competing events. Similarly, for CVDM, deaths due to causes other than CVD are considered competing events. Among other causes of death, there were 546 deaths due to other cancers, 278 deaths from respiratory diseases, 271 from nervous system disorders, and 161 from mental, behavioral, or neurodevelopmental disorders. The primary analyses assume that competing events do not significantly impact the association between lifestyle factors and mortality outcomes. To evaluate this, we applied Fine-Gray models, which account for competing causes of death by including individuals who died from other causes in the risk set. In Fine-Gray models, individuals who die from other causes remain in the risk set for calculating subdistribution hazard ratios (sdHRs), offering a different perspective on competing risks.[16] The results, shown in **eTable 6**, revealed slightly attenuated associations compared to Cox models, but the overall direction of the associations remained consistent. Higher ACS guideline scores were associated with non-significantly lower risks of CVDM (sdHR, 0.98; 95% CI, 0.93 to 1.02) and PCSM (sdHR, 0.95; 95% CI, 0.90 to 1.01) in prostate cancer patients. A similar pattern was observed in patients with non-aggressive tumors. Consistent with Buskova, et al,, where the Fine-Gray model yielded weaker associations, these findings indicate that competing risks modestly influence the results but do not change the overall conclusions.[17] Competing risks, such as deaths from non-cancer causes, reduce the number of individuals at risk of cancer-specific mortality over time. As a result, the cumulative incidence of cancer-specific mortality may appear lower in groups with higher competing risks, regardless of their adherence to the ACS guidelines. This underscores the need for careful interpretation of results in the context of competing risks and the value of using Fine-Gray models to obtain a more comprehensive understanding of the associations.

Sensitivity Analyses

Confounding by Smoking History

We also explored potential confounding by smoking, as it is a known risk factor for mortality and could influence concordance with the ACS guideline.[18,19] Although current smokers at the postdiagnosis survey (n=140) were excluded from the primary analyses, we conducted sensitivity analyses excluding individuals who quit smoking within 10 years of the postdiagnosis survey (n=206).[20] These exclusions did not materially change the results (**eTable 7**), with ACS scores of 6-8 still associated with lower ACM (HR, 0.78; 95% CI, 0.70 to 0.87), CVDM (HR, 0.76; 95% CI, 0.63 to 0.92), and PCSM (HR, 0.84; 95% CI, 0.63 to 1.10). This suggests that recent smoking history did not confound the associations between ACS guideline adherence and mortality.

Reverse Causality

To address concerns about reverse causality, where mortality outcomes could impact concordance with the ACS-guideline at postdiagnosis survey, we conducted analyses excluding outcomes within the first two years (n=155).[21,22] Excluding these early events did not substantially alter the findings (**eTable 8**). ACS guideline scores of 6-8 remained associated with significantly lower ACM (HR, 0.78; 95% CI, 0.70 to 0.86) and CVDM (HR, 0.76; 95% CI, 0.63 to 0.92), suggesting that reverse causation did not significantly affect the estimates, and the protective associations of concordance with the ACS guideline were preserved.

Selection Bias by Conditioning on Non-Missing Postdiagnosis Exposure

1,065 prostate cancer patients were excluded from the analysis due to missing postdiagnosis ACS guideline score. To assess potential selection bias[23] due to conditioning on prostate cancer patients with non-missing postdiagnosis

© 2025 Elahy V et al. *JAMA Network Open*.

assessment of concordance with the ACS guideline (**eFigure 2**), we performed multiple imputations of the missing postdiagnosis ACS guideline score. Missing postdiagnosis ACS guideline scores were imputed using multiple imputation by the Markov Chain Monte Carlo method (MCMC) via PROC MI in SAS.[24,25] Twenty datasets were created using a multivariate normal model, incorporating all of the covariates included in the primary models (race/ethnicity, education, postdiagnosis smoking status and years since quitting, diabetes status, CVD, year of diagnosis, surgical, chemotherapy, and radiation treatment) as well as the mortality outcome. Estimates were pooled using PROC MIANALYZE to obtain final HRs and 95% CIs. The imputation analyses yielded results consistent with the primary findings, showing that higher ACS scores (6-8) were associated with lower ACM (HR, 0.77; 95% CI, 0.70 to 0.85) and CVDM (HR, 0.77; 95% CI, 0.65 to 0.92) (**eTable 9**). These results suggest that conditioning on the participants with non-missing data did not introduce significant selection bias, confirming the robustness of our conclusions.

Proportional Hazards Assumption

Finally, we assessed the proportional hazards assumption by introducing interaction terms between time the and ACS-guideline score used in the primary analysis of the covariates (results not presented). No significant violations were observed, indicating that the hazard ratios are stable over time and can be interpreted as constant across the study period.

© 2025 Elahy V et al. *JAMA Network Open*.

eTable 1. 2022 American Cancer Society (ACS) nutrition and physical activity guideline for cancer survivors

Component	ACS Guideline	Subscore	Subcore criteria
Diet[a]	Follow a healthy eating pattern	0	Lowest tertile of ACS Diet Score
		1	2nd tertile of ACS Diet Score
		2	Highest tertile of ACS Diet Score
Physical Activity	Engage in regular physical activity	0	Moderate to vigorous recreational physical activity <7.5 MET-hrs/wk
		1	Moderate to vigorous recreational physical activity 7.5-15 MET-hrs/wk
		2	Moderate to vigorous recreational physical activity >15 MET-hrs/wk
Body weight	Avoid obesity	0	BMI ≥30 kg/m²
		2	BMI 18.5-<30 kg/m²
Alcohol	Limit alcohol consumption	0	Alcohol intake >2 drinks/day
		1	Alcohol intake >0-≤2 drinks/day
		2	Alcohol intake 0 drinks/day

Abbreviations: ACS guideline, American Cancer Society nutrition and physical activity guideline for cancer survivors; BMI, body mass index; MET-hrs/wk, Metabolic Equivalent of Task hours per week.
[a] See eTable 2 for details of how the ACS Diet score (out of 12) was calculated.

© 2025 Elahy V et al. *JAMA Network Open*.

eTable 2. The American Cancer Society (ACS) Diet score (out of 12) quantifies concordance with the diet component of the ACS Nutrition and Physical Activity Guidelines for Cancer Prevention in the Cancer Prevention Study-II Nutrition Cohort

Component	Survey-specific range[a]			ACS Diet Subscore
	1992	1999	2003	
Fruits (servings/day)[b]	0 –< 0.534	0 – <0.93	0 – <0.970	0
	0.534 – <1.075	0.93 – <1.48	0.970 – <1.58	0.25
	1.075 – <1.713	1.48 – <2.23	1.58 – <2.340	0.50
	≥ 1,713	≥ 2.23	≥ 2.340	0.75
Fruit Variety (varieties/month)[c]	0 – <3	0 – <4	0 – <4	0
	3 – <4	4 – <6	4 – <6	0.25
	4 – <5	6 – <7	6 – <8	0.50
	≥ 5	≥ 7	≥ 8	0.75
Vegetable Intake (servings/day)[d]	0 – <1.2515	0 – <1.750	0 – <1.750	0
	1.2515 – <1.8735	1.750 – <2.640	1.750 – <2.660	0.25
	1.8735 – <2.711	2.640 – <3.870	2.660 – <3.960	0.50
	≥ 2.711	≥ 3.870	≥ 3.960	0.75
Vegetable Variety (varieties/month)[e]	0 – <5	0 – <10	0 – <10	0
	5 – <6	10 – <14	10 – <14	0.25
	6 – <7	14 – <16	14 – <16	0.50
	≥ 7	≥ 16	≥ 16	0.75
Whole Grains (servings/day)[f]	0 – <0.642	0 – <0.50	0 – <0.470	0
	0.642 – <1.35	0.50 – <1.02	0.470 – <1	1
	1.35 – <2.254	1.02 – <2.01	1 – <1.740	2
	≥ 2.254	≥ 2.01	≥ 1.740	3
Red/Processed Meats (servings/day)[g]	0 – <0.391	0 – <0.42	0 – <0.45	3
	0.391 – <0.691	0.42 – <0.67	0.45 – <0.70	2
	0.691 – <1.103	0.67 – <1.05	0.70 – <1.07	1
	≥ 1.103	≥ 1.05	≥ 1.07	0
Sugar-Sweetened Beverages (servings/week)[h]	0	0	0	1.5
	>0 – <3	>0 – <3	>0 – <3	1
	3 – <7	3 – <7	3 – <7	0.5
	≥ 7	≥ 7	≥ 7	0
Highly Processed Foods/Refined Grains (% total energy)[i]	0 – <24.46	0 – <28.69	0 – <26.03	1.5
	24.46 – <31.03	28.69 – <34.49	26.03 – <31.62	1
	31.03 – <37.77	34.49 – <40.68	31.62 – <37.55	0.5
	≥ 37.77	≥ 40.68	≥ 37.55	0

[a] Scoring was done based on sample-specific quartiles.
[b] Total daily servings of fresh, canned, frozen, and dried fruits.
[c] The variety of fruits consumed was measured as the number of distinct types consumed per month.
[d] Total daily servings of fresh, canned, and frozen vegetables.
[e] The variety of vegetables consumed was measured as the number of distinct types of vegetables per month.
[f] Daily servings of whole grain foods: whole grain cereal, cooked oatmeal/oat bran, wheat/oatmeal/other whole grain bread, brown rice, wheat or oat bran added to food, light popcorn, and regular popcorn.

© 2025 Elahy V et al. *JAMA Network Open*.

[g] Daily servings of red and processed meats: bacon, beef/pork hot dogs, chicken/turkey hot dogs, salami, bologna, processed meat sandwiches, sausages, kielbasa, lean hamburger, regular hamburger, beef steak, beef/pork/lamb sandwiches, pork chop, beef/pork/lamb roast, and baked ham. Scoring was reverse ordered, with higher intakes receiving lower scores.

[h] Weekly servings of sugar-sweetened beverages, including carbonated beverages with caffeine and sugar (Coke, Dr. Pepper, Mountain Dew), carbonated beverages with sugar but no caffeine (7-UP), punch, lemonade, other non-carbonated fruit drinks, and sugar-sweetened iced tea.

[i] The percentage of daily calories from highly processed foods and refined grains. These included refined grain cereal, other cooked breakfast cereals, rye/pumpernickel bread, white bread/pita, muffins, bagels/English muffins/pretzels/rolls, pancakes/waffles, white rice, flour tortillas, pasta, nondairy coffee whitener, frozen yogurt, regular ice cream, plain or artificially sweetened yogurt, other flavored yogurt, margarine, salsa/picante/taco sauce, breaded fish cakes/pieces/sticks, French fries, potato or corn chips, fat-free or low-fat crackers, regular crackers/Triscuits/Wheat Thins, low-cal carbonated beverages with caffeine (Diet Coke, Diet Mountain Dew), other low-cal beverages without caffeine (Diet 7-UP), chocolate candy bars, non-chocolate candy, cookies (fat-free, ready-made, or home-baked), brownies, doughnuts, jams/jellies/preserves/syrup/honey, cake (ready-made or home-baked), pies (homemade or ready-made), sweet rolls/coffee cakes/pastries (fat-free or ready-made/home-baked), pizza, pretzels, ketchup/red chili sauce, NutraSweet or Equal, chowder or cream soup, low-fat mayonnaise, regular mayonnaise, and salad dressing. Scoring was reverse ordered, with higher intakes receiving lower scores.

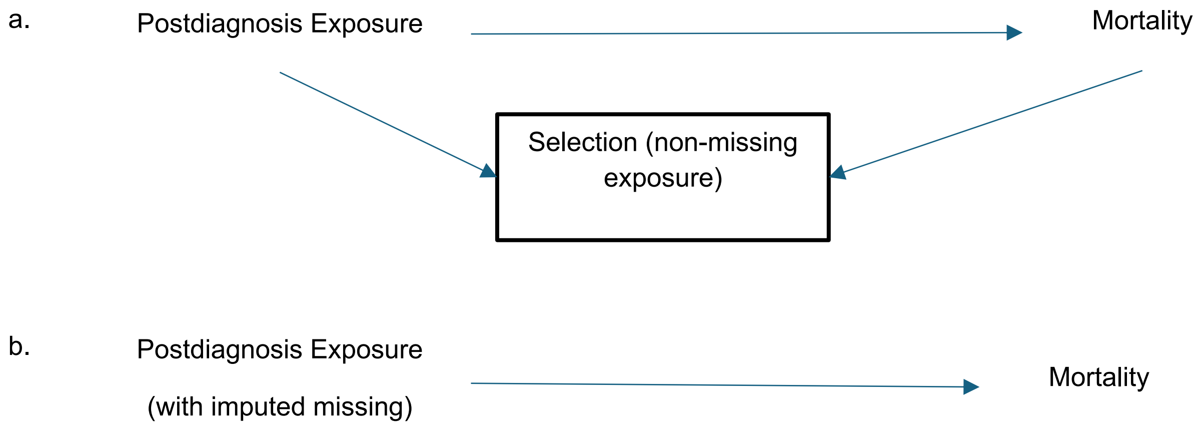

eFigure 1. Directed Acyclic Graph (DAG) illustrating a collider bias and mitigating it using multiple imputation

a. In observational studies examining the effect of postdiagnosis exposure (postdiagnosis American Cancer Society (ACS) guideline concordance) on mortality among cancer survivors, collider bias arises when conditioning on patients with non-missing exposure, which could be partially affected by the outcome of interest. B. Multiple imputation is an attempt to mitigate this bias by imputing missing values of postdiagnosis exposure, thus avoiding the need to condition on selection of non-missing exposures only.

© 2025 Elahy V et al. *JAMA Network Open*.

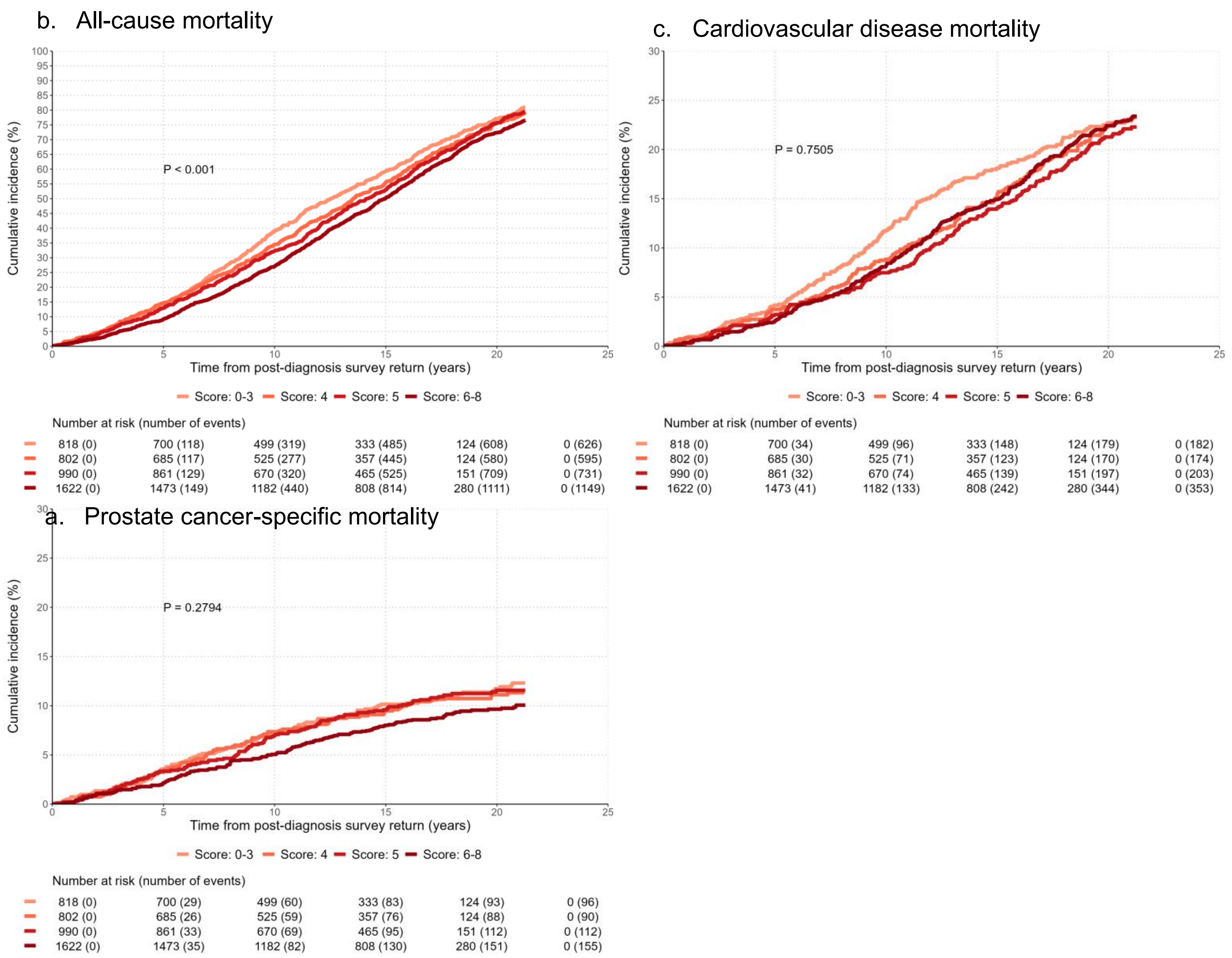

eFigure 2. Cumulative incidence of all-cause, cardiovascular disease-related, and prostate cancer-specific mortality stratified by ACS Guideline score among 4232 prostate cancer patients in the CPS-II Nutrition Cohort (1992-2020).

© 2025 Elahy V et al. *JAMA Network Open*.

eTable 3. Cox Proportional hazard ratios (HRs) and 95% confidence intervals (95% CIs) for the Association of Postdiagnosis individual American Cancer Society (ACS) guideline component scores and mortality among 4,232 patients diagnosed with prostate cancer stratified by prostate cancer aggressiveness in the Cancer Prevention Study-II Nutrition Cohort (1992-2020).

			ACM		CVDM		PCSM	
**ACS Guideline Component**	**Score**	**N**	**Deaths/ Person years**	**HR (95% CI)[a]**	**Deaths/ Person years**	**HR (95% CI)[a]**	**Deaths/ Person years**	**HR (95% CI)[a]**
Prostate cancer patients with non-aggressive tumor[b]								
BMI[c]	0	465	350 / 6,036	1.00 (ref)	105 / 6,036	1.00 (ref)	49 / 6,036	1.00 (ref)
	2	2914	2,087 / 39,135	0.79 (0.71-0.89)	638 / 39,135	0.78 (0.63-0.96)	237 / 39,135	0.64 (0.46-0.88)
Physical activity[d]	0	1109	865 / 13,444	1.00 (ref)	255 / 13,444	1.00 (ref)	113 / 13,444	1.00 (ref)
	1	810	578 / 11,001	0.84 (0.76-0.94)	190 / 11,001	0.95 (0.78-1.15)	62 / 11,001	0.70 (0.51-0.97)
	2	1460	994 / 20,725	0.83 (0.76-0.92)	298 / 20,725	0.88 (0.74-1.05)	111 / 20,725	0.71 (0.54-0.94)
	Per 1-point increase			0.91 (0.87-0.96)		0.94 (0.86-1.02)		0.84 (0.73-0.97)
	P[e]			<0.001		0.15		0.02
Diet[f]	0	1076	796 / 14,160	1.00 (ref)	245 / 14,160	1.00 (ref)	74 / 14,160	1.00 (ref)
	1	1160	834 / 15,387	0.96 (0.87-1.06)	251 / 15,387	0.93 (0.78-1.12)	110 / 15,387	1.41 (1.04-1.92)
	2	1143	807 / 15,624	0.93 (0.84-1.03)	247 / 15,624	0.91 (0.75-1.09)	102 / 15,624	1.47 (1.07-2.01)
	Per 1-point increase			0.97 (0.92-1.02)		0.95 (0.87-1.05)		1.20 (1.03-1.40)
	P[e]			0.19		0.31		0.02
Alcohol[g]	0	459	314 / 6,282	1.00 (ref)	80 / 6,282	1.00 (ref)	47 / 6,282	1.00 (ref)
	1	2232	1,580 / 30,423	0.96 (0.85-1.09)	487 / 30,423	1.14 (0.89-1.45)	182 / 30,423	0.77 (0.55-1.08)
	2	688	543 / 8,466	1.10 (0.95-1.28)	176 / 8,466	1.32 (1.00-1.75)	57 / 8,466	0.72 (0.47-1.09)
	Per 1-point increase			1.07 (0.99-1.15)		1.15 (1.01-1.32)		0.85 (0.69-1.05)
	P[e]			0.10		0.04		0.14
Prostate cancer patients with aggressive tumor[h]								
BMI[c]	0	148	111 / 1,815	1.00 (ref)	28 / 1,815	1.00 (ref)	27 / 1,815	1.00 (ref)
	2	601	464 / 7,308	1.03 (0.82-1.29)	114 / 7,308	1.04 (0.66-1.65)	122 / 7,308	1.28 (0.81-2.03)
Physical activity[d]	0	250	205 / 2,609	1.00 (ref)	64 / 2,609	1.00 (ref)	57 / 2,609	1.00 (ref)
	1	167	128 / 2,071	0.80 (0.63-1.03)	20 / 2,071	0.41 (0.23-0.72)	39 / 2,071	0.93 (0.59-1.45)
	2	332	242 / 4,443	0.75 (0.61-0.92)	58 / 4,443	0.51 (0.34-0.78)	53 / 4,443	0.61 (0.41-0.92)
	Per 1-point increase			0.87 (0.78-0.96)		0.72 (0.58-0.89)		0.78 (0.64-0.96)

© 2025 Elahy V et al. *JAMA Network Open*.

	P[e]			0.007		0.003		0.02
Diet[f]	0	237	187 / 2,810	1.00 (ref)	39 / 2,810	1.00 (ref)	50 / 2,810	1.00 (ref)
	1	240	178 / 2,987	0.89 (0.71-1.11)	46 / 2,987	1.15 (0.72-1.84)	52 / 2,987	0.89 (0.58-1.35)
	2	272	210 / 3,326	0.87 (0.70-1.08)	57 / 3,326	1.15 (0.74-1.81)	47 / 3,326	0.75 (0.49-1.16)
	Per 1-point increase			0.93 (0.84-1.04)		1.07 (0.86-1.33)		0.87 (0.70-1.07)
	P[e]			0.21		0.55		0.19
Alcohol[g]	0	108	76 / 1,339	1.00 (ref)	18 / 1,339	1.00 (ref)	20 / 1,339	1.00 (ref)
	1	478	363 / 5,877	1.02 (0.78-1.34)	91 / 5,877	0.89 (0.50-1.58)	96 / 5,877	1.06 (0.62-1.79)
	2	163	136 / 1,906	1.03 (0.75-1.42)	33 / 1,906	0.69 (0.35-1.36)	33 / 1,906	1.04 (0.56-1.95)
	Per 1-point increase			1.01 (0.87-1.19)		0.82 (0.59-1.14)		1.02 (0.75-1.37)
	P[e]			0.86		0.23		0.92

Abbreviations: ACS guideline, American Cancer Society nutrition and physical activity guideline for cancer survivors; BMI, body mass index; CPS-II, Cancer Prevention Study-II; CVD, cardiovascular disease; CVDM, Cardiovascular disease-related mortality; PCSM, Prostate cancer-specific mortality. Individual component scores were mutually adjusted.

[a] Multivariable models were stratified by single year of age at diagnosis and adjusted for race, education, postdiagnosis smoking status and years since quitting, postdiagnosis diabetes history, postdiagnosis cardiovascular disease history, diagnosis year, surgery, chemotherapy, and radiation therapy. Overall prostate cancer models additionally adjusted for tumor stage, nodal involvement, and Gleason score.

[b] Defined as T1-2 and Gleason score ≤ 7.

[c] Body mass index (BMI) component score of 2 was assigned for a BMI between 18.5-<30 kg/m², and 0 for a BMI of ≥30 kg/m².

[d] Physical Activity component score of 2 was assigned for>15 MET-hrs/wk, 1 for 7.5-15 MET-hrs/wk, and 0 for<7.5 MET-hrs/wk.

[e] P-value for continuous ACS score variable.

[f] Diet component score of 2 was assigned for the highest tertile (≥7.25) of the ACS Diet Score, 1 for the middle tertile (5.25-<7.25), and 0 for the lowest tertile (<5.25).

[g] Alcohol component score of 2 was assigned for 0 drinks/day, 1 for ≤2 drinks/day, and 0 for>2 drinks/day.

[h] Defined as T3-4 or Gleason score>8 or nodal involvement.

© 2025 Elahy V et al. *JAMA Network Open*.

eTable 4. Cox Proportional hazard ratios (HRs) and 95% confidence intervals (95% CIs) for the association of postdiagnosis alternative American Cancer Society (ACS) guideline score (without body mass index [BMI] component) and mortality among 4,232 patients diagnosed with prostate cancer stratified by postdiagnosis BMI in the Cancer Prevention Study-II Nutrition Cohort (1992-2020).

		Alternative ACS Guideline Score (without BMI)					
		**0-2**	**3**	**4**	**5-6**	**Per 1-point increase**	***P*[b]**
**BMI <25 kg/m^2**							
N		380	395	383	361		
ACM	Deaths	295	304	274	253		
	Person Years	4,658	4,999	5,031	5,021		
	HR (95% CI)[a]	1.00 (ref)	1.02 (0.86-1.21)	0.90 (0.75-1.07)	0.80 (0.67-0.95)	0.95 (0.90-0.99)	0.02
CVDM	Deaths	79	89	82	74		
	Person Years	4,658	4,999	5,031	5,021		
	HR (95% CI)[a]	1.00 (ref)	1.06 (0.77-1.45)	0.99 (0.72-1.37)	0.81 (0.58-1.14)	0.97 (0.88-1.05)	0.44
PCSM	Deaths	31	45	34	33		
	Person Years	4,658	4,999	5,031	5,021		
	HR (95% CI)[a]	1.00 (ref)	1.71 (1.04-2.79)	1.10 (0.65-1.86)	1.14 (0.67-1.93)	1.01 (0.88-1.15)	0.91
BMI ≥25 kg/m^2							
N		947	707	632	427		
ACM	Deaths	715	517	427	316		
	Person Years	11,804	9,410	8,810	5,893		
	HR (95% CI)[a]	1.00 (ref)	0.92 (0.82-1.04)	0.77 (0.68-0.87)	0.89 (0.78-1.02)	0.94 (0.91-0.97)	<0.001
CVDM	Deaths	214	149	134	91		
	Person Years	11,804	9,410	8,810	5,893		
	HR (95% CI)[a]	1.00 (ref)	0.91 (0.73-1.13)	0.79 (0.63-0.99)	0.86 (0.67-1.11)	0.93 (0.88-1.00)	0.03
PCSM	Deaths	128	73	58	51		
	Person Years	11,804	9,410	8,810	5,893		
	HR (95% CI)[a]	1.00 (ref)	0.73 (0.54-0.98)	0.61 (0.44-0.85)	0.87 (0.62-1.21)	0.90 (0.83-0.98)	0.02

Abbreviations: ACS guideline, American Cancer Society nutrition and physical activity guideline for cancer survivors; BMI, body mass index; CPS-II, Cancer Prevention Study-II; CVDM, Cardiovascular disease-related mortality; PCSM, Prostate cancer-specific mortality.

[a] Multivariable models were stratified by single year of age at diagnosis and adjusted for race, education, postdiagnosis smoking status and years since quitting, postdiagnosis diabetes history, postdiagnosis cardiovascular disease history, diagnosis year, surgery, chemotherapy, and radiation therapy. Overall prostate cancer models additionally adjusted for tumor stage, nodal involvement, and Gleason score.

[b] P-value for trend analyses across the continuous alternative ACS Guideline score variable.

© 2025 Elahy V et al. *JAMA Network Open*.

eTable 5. Cox Proportional hazard ratios (HRs) and 95% confidence intervals (95% CIs) for the association of postdiagnosis total American Cancer Society (ACS) guideline score and mortality among 4,232 patients diagnosed with prostate cancer stratified by prevalent cardiovascular disease reported at postdiagnosis survey in the Cancer Prevention Study-II Nutrition Cohort (1992-2020).

		ACS Guideline Score					
		**0-3**	**4**	**5**	**6-8**	**Per 1-point increase**	***P***[b]
**No CVD**							
N		547	529	699	1,151		
ACM	Deaths	399	356	480	764		
	Person Years	7,200	7,314	9,758	16,593		
	HR (95% CI)[a]	1.00 (ref)	0.79 (0.68-0.91)	0.79 (0.69-0.91)	0.73 (0.64-0.83)	0.93 (0.90-0.96)	<0.001
CVDM	Deaths	103	81	114	229		
	Person Years	7,200	7,314	9,758	16,593		
	HR (95% CI)[a]	1.00 (ref)	0.65 (0.48-0.87)	0.68 (0.52-0.90)	0.78 (0.61-1.00)	0.95 (0.90-1.01)	0.11
PCSM	Deaths	64	62	77	100		
	Person Years	7,200	7,314	9,758	16,593		
	HR (95% CI)[a]	1.00 (ref)	1.01 (0.70-1.45)	0.98 (0.69-1.40)	0.73 (0.53-1.02)	0.90 (0.84-0.97)	0.005
**CVD**							
N		271	273	291	471		
ACM	Deaths	227	239	251	385		
	Person Years	2,947	2,960	3,212	5,643		
	HR (95% CI)[a]	1.00 (ref)	1.02 (0.84-1.24)	1.07 (0.88-1.29)	0.82 (0.69-0.97)	0.95 (0.91-0.98)	0.005
CVDM	Deaths	79	93	89	124		
	Person Years	2,947	2,960	3,212	5,643		
	HR (95% CI)[a]	1.00 (ref)	1.07 (0.78-1.48)	1.00 (0.72-1.38)	0.67 (0.50-0.91)	0.90 (0.84-0.96)	0.002
PCSM	Deaths	32	28	35	55		
	Person Years	2,947	2,960	3,212	5,643		
	HR (95% CI)[a]	1.00 (ref)	0.84 (0.48-1.45)	1.01 (0.60-1.69)	0.89 (0.56-1.42)	0.99 (0.89-1.10)	0.87

Abbreviations: ACS guideline, American Cancer Society nutrition and physical activity guideline for cancer survivors; CPS-II, Cancer Prevention Study-II; CVD, cardiovascular disease; CVDM, Cardiovascular disease-related mortality; PCSM, Prostate cancer-specific mortality.

[a] Multivariable models were stratified by single year of age at diagnosis and adjusted for race, education, postdiagnosis smoking status and years since quitting, postdiagnosis diabetes history, diagnosis year, surgery, chemotherapy, and radiation therapy. Overall prostate cancer models additionally adjusted for tumor stage, nodal involvement, and Gleason score.

[b] P-value for trend analyses across the continuous ACS score variable.

eTable 6. Subdistribution hazard ratios (sdHR) and 95% confidence intervals (95% CI) for the association of postdiagnosis total American Cancer Society (ACS) guideline score and CVDM and PCSM among 4,232 patients diagnosed with prostate cancer in the Cancer Prevention Study-II Nutrition Cohort (1992-2020).

		**ACS Guideline Score**					
		**0-3**	**4**	**5**	**6-8**	**Per 1-point increase**	***P*[b]**
N		818	802	990	1622		
CVDM	Deaths	182	174	203	353		
	Person Years	10,147	10,274	12,970	22,236		
	sdHR (95% CI)[a]	1.00 (ref)	0.89 (0.72-1.10)	0.85 (0.69-1.05)	0.90 (0.74-1.08)	0.98 (0.93-1.02)	0.28
PCSM	Deaths	96	90	112	155		
	Person Years	10,147	10,274	12,970	22,236		
	sdHR (95% CI)[a]	1.00 (ref)	1.01 (0.75-1.35)	1.03 (0.78-1.37)	0.86 (0.66-1.12)	0.95 (0.90-1.01)	0.12

Abbreviations: ACS guideline, American Cancer Society nutrition and physical activity guideline for cancer survivors; CPS-II, Cancer Prevention Study-II; CVDM, Cardiovascular disease-related mortality; PCSM, Prostate cancer-specific mortality.

[a] Subdistribution hazard ratios (sdHR) are produced using Fine-Gray models accounting for competing causes of death. Models were stratified by single year of age at diagnosis and adjusted for race, education, postdiagnosis smoking status and years since quitting, postdiagnosis diabetes history, postdiagnosis cardiovascular disease history, diagnosis year, surgery, chemotherapy, and radiation therapy. Overall prostate cancer models additionally adjusted for tumor stage, nodal involvement, and Gleason score.

[b] P-value for trend analyses across the continuous ACS Guideline score variable.

© 2025 Elahy V et al. *JAMA Network Open*.

eTable 7. Cox Proportional hazard ratios (HRs) and 95% confidence intervals (95% CIs) for the association of postdiagnosis total American Cancer Society (ACS) guideline score and mortality among 4,026 patients diagnosed with prostate cancer, excluding those who quit smoking within 10 years of postdiagnosis questionnaire return in the Cancer Prevention Study-II Nutrition Cohort (1992-2020).

		ACS Guideline Score					
		**0-3**	**4**	**5**	**6-8**	**Per 1-point increase**	***P*[b]**
N		754	753	945	1574		
ACM	Deaths	571	553	694	1,111		
	Person Years	9,417	9,707	12,453	21,583		
	HR (95% CI)[a]	1.00 (ref)	0.87 (0.77-0.98)	0.86 (0.77-0.97)	0.78 (0.70-0.87)	0.94 (0.92-0.96)	<0.001
CVDM	Deaths	168	165	191	340		
	Person Years	9,417	9,707	12,453	21,583		
	HR (95% CI)[a]	1.00 (ref)	0.83 (0.67-1.04)	0.78 (0.63-0.96)	0.76 (0.63-0.92)	0.93 (0.89-0.98)	0.002
PCSM	Deaths	84	86	109	152		
	Person Years	9,417	9,707	12,453	21,583		
	HR (95% CI)[a]	1.00 (ref)	1.01 (0.74-1.38)	1.03 (0.77-1.38)	0.84 (0.63-1.10)	0.94 (0.88-1.00)	0.04

Abbreviations: ACS guideline, American Cancer Society nutrition and physical activity guideline for cancer survivors; CPS-II, Cancer Prevention Study-II; CVDM, Cardiovascular disease-related mortality; PCSM, Prostate cancer-specific mortality.

[a] Multivariable models were stratified by single year of age at diagnosis and adjusted for race, education, postdiagnosis smoking status and years since quitting, postdiagnosis diabetes history, postdiagnosis cardiovascular disease history, diagnosis year, surgery, chemotherapy, and radiation therapy. Overall prostate cancer models additionally adjusted for tumor stage, nodal involvement, and Gleason score.

[b] P-value for trend analyses across the continuous ACS Guideline score variable.

eTable 8. Cox Proportional hazard ratios (HRs) and 95% confidence intervals (95% CIs) for the association of postdiagnosis total American Cancer Society (ACS) guideline score and mortality among 4,077 patients diagnosed with prostate cancer, excluding outcome events that occurred within the first 2 years of follow-up to account for reverse causality in the Cancer Prevention Study-II Nutrition Cohort (1992-2020).

		ACS Guideline Score					
		**0-3**	**4**	**5**	**6-8**	**Per 1-point increase**	***P*[b]**
N		782	765	951	1579		
ACM	Deaths	590	558	692	1,106		
	Person Years	8,545	8,705	11,024	19,034		
	HR (95% CI)[a]	1.00 (ref)	0.86 (0.77-0.97)	0.86 (0.77-0.97)	0.78 (0.70-0.86)	0.94 (0.92-0.96)	<0.001
CVDM	Deaths	172	163	194	342		
	Person Years	8,545	8,705	11,024	19,034		
	HR (95% CI)[a]	1.00 (ref)	0.81 (0.65-1.01)	0.79 (0.64-0.98)	0.76 (0.63-0.92)	0.93 (0.89-0.98)	0.003
PCSM	Deaths	85	84	101	139		
	Person Years	8,545	8,705	11,024	19,034		
	HR (95% CI)[a]	1.00 (ref)	1.01 (0.74-1.38)	0.98 (0.73-1.33)	0.79 (0.60-1.05)	0.93 (0.87-0.99)	0.03

Abbreviations: ACS guideline, American Cancer Society nutrition and physical activity guideline for cancer survivors; CPS-II, Cancer Prevention Study-II; CVDM, Cardiovascular disease-related mortality; PCSM, Prostate cancer-specific mortality.

[a] Multivariable models were stratified by single year of age at diagnosis and adjusted for race, education, postdiagnosis smoking status and years since quitting, postdiagnosis diabetes history, postdiagnosis cardiovascular disease history, diagnosis year, surgery, chemotherapy, and radiation therapy. Overall prostate cancer models additionally adjusted for tumor stage, nodal involvement, and Gleason score.

[b] P-value for trend analyses across the continuous ACS Guideline score variable.

© 2025 Elahy V et al. *JAMA Network Open*.

eTable 9. Cox Proportional hazard ratios (HRs) and 95% confidence intervals (95% CIs) for the association of postdiagnosis total American Cancer Society (ACS) guideline score and mortality among 5,297 patients diagnosed with prostate cancer where missing postdiagnosis ACS guideline score were imputed[b] if missing in the Cancer Prevention Study-II Nutrition Cohort (1992-2020).

	ACS Guideline Score					
	**0-3**	**4**	**5**	**6-8**	**Per 1-point increase**	***P*[c]**
ACM	1.00 (ref)	0.95 (0.85-1.05)	0.87 (0.79-0.96)	0.77 (0.70-0.85)	0.94 (0.92-0.96)	<0.001
CVDM	1.00 (ref)	0.96 (0.80-1.16)	0.83 (0.68-1.01)	0.77 (0.65-0.92)	0.93 (0.90-0.97)	<0.001
PCSM	1.00 (ref)	0.99 (0.73-1.33)	0.91 (0.69-1.21)	0.75 (0.57-0.98)	0.93 (0.87-0.98)	0.009

Abbreviations: ACS guideline, American Cancer Society nutrition and physical activity guideline for cancer survivors; CPS-II, Cancer Prevention Study-II; CVDM, Cardiovascular disease-related mortality; PCSM, Prostate cancer-specific mortality.

[a] Multivariable models were stratified by single year of age at diagnosis and adjusted for race, education, postdiagnosis smoking status and years since quitting, postdiagnosis diabetes history, postdiagnosis cardiovascular disease history, diagnosis year, surgery, chemotherapy, and radiation therapy. Overall prostate cancer models additionally adjusted for tumor stage, nodal involvement, and Gleason score.

[b] Multiple imputations were performed to account for missing ACS Guideline score values. 20 datasets were created with missing values replaced by imputed values based on a model that incorporated demographic and clinical variables. The results of these datasets were then combined using Rubin's rules.

[c] P-value for trend analyses across the continuous ACS score variable.

© 2025 Elahy V et al. *JAMA Network Open*.

eReferences

1. Mohler J, Bahnson RR, Boston B, et al. Prostate Cancer. *Journal of the National Comprehensive Cancer Network*. 2010;8(2):162-200. doi:10.6004/jnccn.2010.0012
2. Calle EE, Rodriguez C, Jacobs EJ, et al. The American Cancer Society Cancer Prevention Study II Nutrition Cohort. *Cancer*. 2002;94(2):500-511. doi:10.1002/cncr.10197
3. Rees-Punia E, Patel AV, Nocera JR, et al. Self-reported physical activity, sitting time, and mental and physical health among older cancer survivors compared with adults without a history of cancer. *Cancer*. 2021;127(1):115-123. doi:10.1002/cncr.33257
4. Herrmann SD, Willis EA, Ainsworth BE, et al. 2024 Adult Compendium of Physical Activities: A third update of the energy costs of human activities. *Journal of Sport and Health Science*. 2024;13(1):6-12. doi:10.1016/j.jshs.2023.10.010
5. Patel AV, Carter BD, Stevens VL, Gaudet MM, Campbell PT, Gapstur SM. The relationship between physical activity, obesity, and lung cancer risk by smoking status in a large prospective cohort of US adults. *Cancer Causes & Control*. 2017;28(12):1357-1368. doi:10.1007/s10552-017-0949-0
6. Flagg EW, Coates RJ, Calle EE, Potischman N, Thun MJ. Validation of the American Cancer Society Cancer Prevention Study II Nutrition Survey Cohort Food Frequency Questionnaire. *Epidemiology*. 2000;11(4):462-468. doi:10.1097/00001648-200007000-00017
7. Feskanich D, Rimm EB, Giovannucci EL, et al. Reproducibility and validity of food intake measurements from a semiquantitative food frequency questionnaire. *Journal of the American Dietetic Association*. 1993;93(7):790-796. doi:10.1016/0002-8223(93)91754-e
8. McCullough ML, Chantaprasopsuk S, Islami F, et al. Association of Socioeconomic and Geographic Factors With Diet Quality in US Adults. *JAMA Network Open*. 2022;5(6):e2216406. doi:10.1001/jamanetworkopen.2022.16406
9. Farris MS, Courneya KS, Kopciuk KA, McGregor SE, Friedenreich CM. Post-diagnosis alcohol intake and prostate cancer survival: A population-based cohort study. *International Journal of Cancer*. 2018;143(2):253-262. doi:10.1002/ijc.31307
10. Kenfield SA, Stampfer MJ, Giovannucci E, Chan JM. Physical Activity and Survival After Prostate Cancer Diagnosis in the Health Professionals Follow-Up Study. *Journal of Clinical Oncology*. 2011;29(6):726-732. doi:10.1200/jco.2010.31.5226
11. Dickerman BA, Giovannucci E, Pernar CH, Mucci LA, Hernán MA. Guideline-Based Physical Activity and Survival Among US Men With Nonmetastatic Prostate Cancer. *American Journal of Epidemiology*. 2019;188(3):579-586. doi:10.1093/aje/kwy261
12. Campbell KL, Winters-Stone KM, Wiskemann J, et al. Exercise Guidelines for Cancer Survivors: Consensus Statement from International Multidisciplinary Roundtable. *Medicine & Science in Sports & Exercise*. 2019;51(11):2375-2390. doi:10.1249/mss.0000000000002116
13. Coletta AM, Simon LH, Maslana K, et al. Creatine supplementation and resistance training to preserve muscle mass and attenuate cancer progression (CREATINE-52): a protocol for a double-blind randomized controlled trial. *BMC Cancer*. 2024;24(1)doi:10.1186/s12885-024-12260-3
14. Troeschel AN, Hartman TJ, Jacobs EJ, et al. Postdiagnosis Body Mass Index, Weight Change, and Mortality From Prostate Cancer, Cardiovascular Disease, and All Causes Among Survivors of Nonmetastatic Prostate Cancer. *Journal of Clinical Oncology*. 2020;38(18):2018-2027. doi:10.1200/jco.19.02185
15. Kenfield SA, DuPre N, Richman EL, Stampfer MJ, Chan JM, Giovannucci EL. Mediterranean diet and prostate cancer risk and mortality in the Health Professionals Follow-up Study. *European urology*. 2014;65(5):887-894.
16. Fine JP, Gray RJ. A Proportional Hazards Model for the Subdistribution of a Competing Risk. *Journal of the American Statistical Association*. 1999/06/01 1999;94(446):496-509. doi:10.1080/01621459.1999.10474144
17. Buzkova P, Barzilay JI, Mukamal KJ. Assessing risk factors of non-fatal outcomes amid a competing risk of mortality: the example of hip fracture. *Osteoporosis International*. 2019;30(10):2073-2078. doi:10.1007/s00198-019-05048-w
18. Stokes A, Preston SH. Smoking and reverse causation create an obesity paradox in cardiovascular disease. *Obesity*. 2015;23(12):2485-2490. doi:10.1002/oby.21239
19. Bigaard J, Tjønneland A, Thomsen BL, Overvad K, Heitmann BL, Sørensen TIA. Waist Circumference, BMI, Smoking, and Mortality in Middle-Aged Men and Women. *Obesity Research*. 2003;11(7):895-903. doi:10.1038/oby.2003.123
20. Cho ER, Brill IK, Gram IT, Brown PE, Jha P. Smoking Cessation and Short- and Longer-Term Mortality. *NEJM Evidence*. 2024;3(3)doi:10.1056/evidoa2300272

© 2025 Elahy V et al. *JAMA Network Open*.

21. Rezende LFM, Lee DH, Giovannucci E. Possible Reverse Causation and Confounding in Study of the Association of Sedentary Behavior With Cancer Mortality. *JAMA Oncology*. 2021;7(1):138. doi:10.1001/jamaoncol.2020.5874
22. Garcia GR, Coleman NC, Pond ZA, Pope CA. Shape of BMI–Mortality Risk Associations: Reverse Causality and Heterogeneity in a Representative Cohort of US Adults. *Obesity*. 2021;29(4):755-766. doi:10.1002/oby.23114
23. Infante-Rivard C, Cusson A. Reflection on modern methods: selection bias—a review of recent developments. *International Journal of Epidemiology*. 2018;47(5):1714-1722. doi:10.1093/ije/dyy138
24. Hernán MA, Hernández-Díaz S, Robins JM. A Structural Approach to Selection Bias. *Epidemiology*. 2004;15(5):615-625. doi:10.1097/01.ede.0000135174.63482.43
25. Rubin D. Multiple Imputation for Nonresponse in Surveys. New York, NY: JohnWiley & Sons. Inc; 1987.

© 2025 Elahy V et al. *JAMA Network Open*.
