# Supplementary material for: Adherence to American Cancer Society Guideline and Mortality in Men With Nonmetastatic Prostate Cancer
Source: JAMA Netw Open. 2025 Sep 26;8(9):e2533922. doi: 10.1001/jamanetworkopen.2025.33922 (PMC12475947; doi:10.1001/jamanetworkopen.2025.33922)
Supplement: Supplement 2. — Data Sharing Statement [file jamanetwopen-e2533922-s002.pdf]

Data Sharing Statement

Data
Data available: Yes
Data types: Deidentified participant data, Data dictionary
How to access data: Please email cohort.data@cancer.org to inquire about access.
When available: With publication

Supporting Documents
Document types: None

Additional Information
Who can access the data: Data are available from the American Cancer Society by following the ACS Data Access Procedures (https://www.cancer.org/research/population-science/research-collaboration.html) for researchers who meet the criteria for access to confidential data.
Types of analyses: For a specified purpose
Mechanisms of data availability: After approval of a proposal with a signed data access agreement
Any additional restrictions: NA
